# Deficiency of osteoblastic Arl6ip5 impaired osteoblast differentiation and enhanced osteoclastogenesis via disturbance of ER calcium homeostasis and induction of ER stress-mediated apoptosis

**DOI:** 10.1038/cddis.2014.427

**Published:** 2014-10-16

**Authors:** Y Wu, M Yang, J Fan, Y Peng, L Deng, Y Ding, R Yang, J Zhou, D Miao, Q Fu

**Affiliations:** 1 Key Laboratory of Nuclear Medicine, Ministry of Health, Jiangsu Key Laboratory of Molecular Nuclear Medicine, Jiangsu Institute of Nuclear Medicine, Wuxi, China; 2 Department of Radiotherapy, The First Affiliated Hospital of Nanjing Medical University, Nanjing, China; 3 Department of Molecular Cell Biology and Toxicology, School of Public Health, Nanjing Medical University, Nanjing, China; 4 State Key Laboratory of Reproductive Medicine, The Research Center for Bone and Stem Cells, Department of Anatomy, Histology, and Embryology, Nanjing, China

## Abstract

ADP-ribosylation-like factor 6 interacting protein 5 (Arl6ip5), which belongs to the prenylated rab-acceptor-family, has an important role in exocytic protein trafficking, glutathione metabolism and involves in cancer progression. However, its expression pattern and functional role in bone are unknown. Here we demonstrate that Arl6ip5 knock-out mice (Arl6ip5 ^*Δ2/Δ2*^) show marked decrease of bone mineral density, trabecular bone volume and trabecular thickness. Histomorphometric studies reveal that bone formation parameters are decreased but bone resorption parameters and mRNA level of osteoclast-specific markers are increased in Arl6ip5^*Δ2/Δ2*^ mice. In osteoblast, we demonstrate that Arl6ip5 abundantly expresses in osteoblastic cells and is regulated by bone metabolism-related hormones and growth factors. *In vitro* analysis reveals that osteoblast proliferation and differentiation are impaired in Arl6ip5 knocked-down and deficient primary osteoblast. Arl6ip5 is also found to function as an ER calcium regulator and control calmodulin signaling for osteoblast proliferation. Moreover, Arl6ip5 insufficiency in osteoblast induces ER stress and enhances ER stress-mediated apoptosis. CCAAT/enhancer-binding protein homologous protein (Chop) is involved in the regulation of apoptosis and differentiation in Arl6ip5 knocked-down osteoblasts. For osteoclastogenesis, Arl6ip5 insufficiency in osteoclast precursors has no effect on osteoclast formation. However, knocked-down osteoblastic Arl6ip5 induces receptor activator of nuclear factor-*κ*B ligand (RANKL) expression and enhances osteoclastogenesis. In addition, ER stress and Chop are involved in the RANKL expression in Arl6ip5 knocked-down osteoblasts. In conclusion, we demonstrate that Arl6ip5 is a novel regulator of bone formation in osteoblasts.

Bone is a dynamic tissue that undergoes constant remodeling throughout life.^1^ Bone remodeling is a complex process involving the removal of mineralized bone by osteoclasts and the formation of bone matrix through osteoblasts. Osteoclasts, derived from hematopoietic stem cells, control bone resorption. Two cytokines, receptor activator of nuclear factor-*κ*B ligand (RANKL) and macrophage colony-stimulating factor (M-CSF), are necessary for the proliferation and differentiation of osteoclast precursors, as well as the maintenance of survival and activation of osteoclasts.^1^ Mature osteoblasts are differentiated from mesenchymal progenitors via a set of distinct cellular intermediates.^2^ The process of cellular differentiation from mesenchymal progenitors to osteoblast is modulated by a network of signaling pathways and Runx2 and osterix transcription factors.^2,3^ Osteoblast proliferation, survival and differentiation are also regulated by some intracellular events, such as calcium signaling and unfolded protein response (UPR).^4, 5, 6^

Calcium (Ca^2+^) is an essential intracellular signaling molecule involved in the regulation of numerous cellular processes including cell proliferation, differentiation, morphology and function.^7^ In resting cell, the concentration of intracellular Ca^2+^ ([Ca^2+^]_i_) is maintained in very low level and the cytoplasmic Ca^2+^ is actively pumped from cytosol into endoplasmic reticulum (ER), an intracellular Ca^2+^ storage organelle, or extruded by Ca^2+^ transport systems to the extracellular space. [Ca^2+^]_i_ can be suddenly raised via Ca^2+^ influx from the extracellular space or Ca^2+^ release from ER.^8^ The release of Ca^2+^ from ER is mainly regulated by the inositol trisphosphate 3 (IP3) receptors (IP3Rs) in unexcitable cells and ryanodine receptors (RyRs) in excitable cells.^9^ Calcium activates signaling pathway via its binding protein calmodulin (CaM) and downstream kinases. The CaM-CaMKII (Ca^2+^/CaM-dependent protein kinase II, CaMKII) pathway has been demonstrated to regulate osteoblast proliferation and differentiation.^4,10^ In addition, Ca^2+^ is also involved in the synthesis, folding and post-translational modifications of proteins in ER. Disturbance of Ca^2+^ balance activates UPR that attempts to restore the homeostasis.^11^

UPR, with its ability to sense the insufficiency of protein folding in ER and communicate this information to gene expression programs, has critical roles in the establishment and maintenance for cellular homeostasis, especially in highly secreting cells, such as osteoblasts that produce many important factors for bone formation and bone resorption.^6,12^ Usually, the unfolded protein stress in ER (ER stress) activates the ATF6, IRE1 and PERK – branches of UPR and regulates the expression of target genes involved in the modulation of ER protein folding, such as Bip, Grp94 and XBP1.^12^ The deficiency of UPR signaling proteins such as activating transcription factor 4 (ATF4) and OASIS lead to aberrant bone development and bone loss phenotype in mice.^13,14^ It has been demonstrated that mild ER stress is helpful for osteoblast differentiation.^5^ However, if the stress prolonged and unmitigated, the UPR switches over to initiate cell apoptosis, which is largely mediated by the ATF4-CCAAT/enhancer-binding protein homologous protein (CHOP)-GADD34 signaling axis.^15,16^ It is still not clear whether this persistent stress in osteoblast would lead to apoptosis and affect the communication between osteoblast and other bone cells such as osteoclast.

ADP-ribosylation-like factor 6 interacting protein 5 (Arl6ip5, synonym JWA), which belongs to the prenylated rab-acceptor-family, ubiquitously expresses in various tissues and is induced by diversity of stimuli such as ER Ca^2+^-depletion, heat shock and oxidative stress.^17, 18, 19^ A microarray study demonstrated that the Arl6ip5 is one of the genes regulated by the parathyroid hormone (PTH) and PTHrP in osteoblasts.^20^ In our previous study, we constructed Arl6ip5 conditional knockout (KO) mice and found some bone-related phenotypes such as kyphosis, ^21^ but its exact role in bone metabolism and the underlying mechanism are largely unknown.

In this study, we investigate the role of Arl6ip5 in bone with an exquisite analysis for the bone metabolism-related parameters and find that Arl6ip5 KO in mice lead to bone loss. We also find that Arl6ip5 is an ER localized protein in osteoblast and is regulated by osteotropic factors. Arl6ip5 insufficiency in osteoblast disturbs calcium homeostasis, induced ER stress-mediated apoptosis and impairs osteoblast proliferation and differentiation. Moreover, Arl6ip5 insufficiency indirectly enhances the osteoclastogenesis through increasing RANKL expression in osteoblast.

## Results

### Arl6ip5 deficiency induces bone loss phenotype in mice

**Figure 1:**
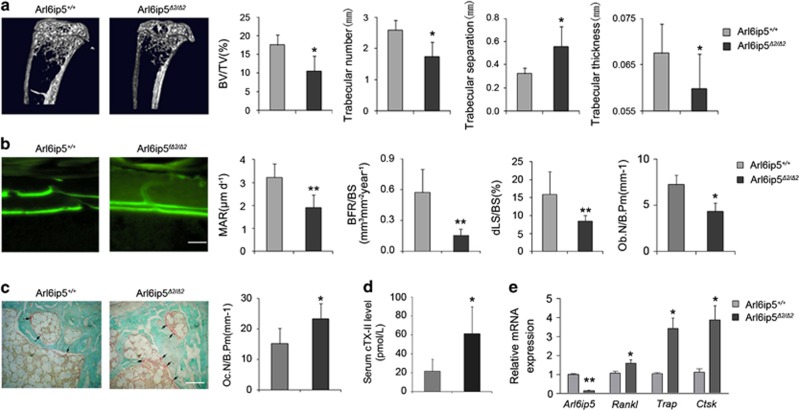
Arl6ip5^*Δ2/Δ2*^ mice show bone loss phenotype. (**a**) *μ*-CT images and three-dimensional microstructural analysis of tibias from 16-week-old Arl6ip5^+/+^ (*n*=10), Arl6ip5^*Δ2/Δ2*^ (*n*=12) mice. Bone volume per tissue volume (BV/TV, %). (**b**) Dynamic bone histomorphometry and osteoblast number (Ob.N) of Arl6ip5^+/+^ (*n*=6) and Arl6ip5^*Δ2/Δ2*^ mice (*n*=6). (**c**) TRAP staining images of the tibias from 16-week-old Arl6ip5^+/+^ (*n*=6), Arl6ip5^*Δ2/Δ2*^ (*n*=6) mice. Scale bar, 100 *μ*m. (**d**) The serum level of cTx-II in Arl6ip5^+/+^ (*n*=6) and Arl6ip5^*Δ2/Δ2*^ mice (*n*=6). (**e**) Expression of *Arl6ip5*, *RANKL*, *Trap* and *Ctsk* mRNAs in the tibias from Arl6ip5^*Δ2/Δ2*^ (*n*=6) and Arl6ip5^+/+^ (*n*=6) mice. In all panels, bar represents mean±S.E.M., *P<0.05, **P<0.01. All *P-*values were based on *Student*'s *t*-test

Arl6ip5 is a widely expressed protein. Here, we found that Arl6ip5 mRNA also expressed in bone tissues lumbar vertebra, tibia, femur and calvaria (Supplementary Figure S1). To assess the *in vivo* role of Arl6ip5, we constructed the Arl6ip5 deficiency mice with Arl6ip5 exon2 deletion in whole body (Arl6ip5^*Δ2/Δ2*^ mice)^21^ and found these mice with growth retardation and severe scoliosis, which were not observed in Arl6ip5^*+/+*^ mice. The micro-computed tomography (*μ*-CT) analysis showed that the bone mineral density of tibia and vertebra were 10–15% reduced in Arl6ip5^*Δ2/Δ2*^ mice compared with control littermates at 4 months of age (Figure 1a and Supplementary Figure S2), which was observed in both genders (data not shown). Quantitative analyses further demonstrated that 40% less of BV/TV (*P*<0.05) and significant decrease of trabecular thickness (Tb.Th) (*P*<0.05) and trabecular number (Tb.N) (*P*<0.05) but significant increase of trabecular separation (Tb.Sp) (*P*<0.05) in Arl6ip5^*Δ2/Δ2*^ mice compared with Arl6ip5^+/+^ mice (Figure 1a). However, no distinction was found in levels of serum calcium, phosphate, glucose, albumin and cholesterol between Arl6ip5^*Δ2/Δ2*^ mice and Arl6ip5^+/+^ mice (data not shown).

Dynamic histomorphometry of the proximal tibia revealed that bone formation parameters such as mineral apposition rate (MAR, *P*<0.01), BFR/BS (*P*<0.01) and dLS/BS (*P*<0.01) of cortical bone (Figure 1b) and MAR of trabecular bone (Supplementary Figure S3) were significantly decreased in Arl6ip5^*Δ2/Δ2*^ mice compared with control mice at 4 months of age. Histological analysis further revealed a significant decrease in osteoblasts number (*P*<0.05) (Figure 1b) and an increase in the number of tartrate-resistant acid phosphatase (TRAP)-positive osteoclasts (*P*<0.05) in the proximal tibia of Arl6ip5^*Δ2/Δ2*^ mice compared with Arl6ip5^+/+^ mice (Figure 1c). In consistence, the serum level of cTX-II (Figure 1d) and mRNA expression of *RANKL* (1.49-fold, *P*<0.05), *Trap* (3.35-fold, *P*<0.05) and *Ctsk* (3.45-fold, *P*<0.05) (Figure 1e) in the tibia of Arl6ip5^*Δ2/Δ2*^ mice were also significant higher than that in control mice.

### Arl6ip5 localizes in ER and is stimulated by osteotropic factors in osteoblast

**Figure 2:**
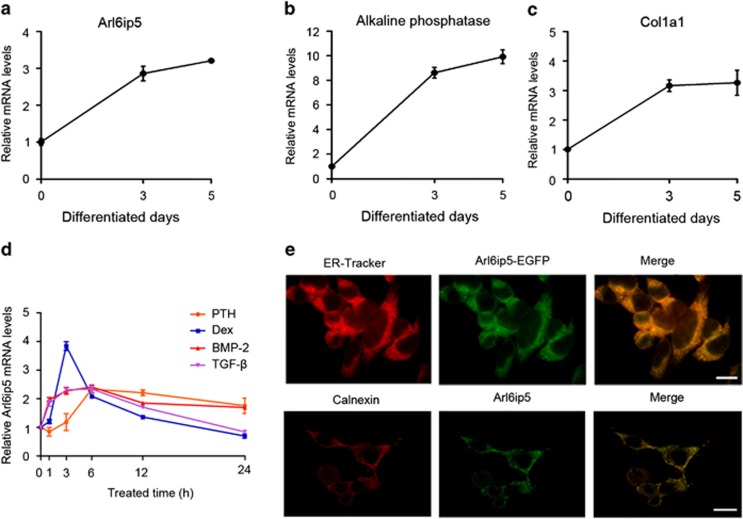
The basic and induced expression pattern of Arl6ip5 in osteoblasts. (**a**–**c**) UAMS-32 cells were treated with BMP-2 (100 ng/ml) for the indicated days to induce differentiation, then mRNA level of Arl6ip5 (**a**), alkaline phosphatase (**b**) and Col1a1(**c**) was analyzed with Q-PCR. *n*=3. In all panels, bars represent mean±S.E.M. (**d**) Arl6ip5 mRNA levels detected by Q-PCR analysis after treating UAMS-32 cells with indicated dosage of PTH (100 nM), Dex (400 nM), TGF*β*1 (10 ng/ml) and BMP-2 (100 ng/ml) for 0, 1, 3, 6, 12 and 24 h. *n*=4. (**e**) Arl6ip5-EGFP was transiently transfected into POBs and its localization with ER-specific indicator (ER-tracker) was analyzed with fluorescence microscope (upper panel). Immunofluorescence staining with the antibodies to Arl6ip5 and ER marker calnexin was used to analyze the localization of endogenous Arl6ip5 to ER (lower panel). Scale bar=10 *μ*m

To understand the role of Arl6ip5 in osteoblasts, the mRNA level and subcellular localization of Arl6ip5 were determined in primary calvarial osteoblasts (POBs) and stromal/osteoblast cell line (UAMS-32). We found that Arl6ip5 mRNA expressed in bone marrow cells, POBs and osteoblast cell line (data not shown). For bone marrow cells, the mRNA level of Arl6ip5 in adherent cells was significantly higher than that in non-adherent cells (Supplementary Figure S4). In the differentiated UAMS-32 cells induced by bone morphogenetic protein 2 (BMP-2), as identified by the enhancing expression of specific osteoblast differentiation markers alkaline phosphatase (ALP) and Col1a1, the expression of Arl6ip5 was increased (Figures 2a–c). In UAMS-32 cells, the expression of Arl6ip5 was quickly upregulated by osteotropic factors (Figure 2d). The peak level of Arl6ip5 expression was at 3 h for dexamethasone (Dex) treatment (3.83-fold, *P*<0.05) and at 6 h for transforming growth factor *β*1 (TGF-*β*1; 2.34-fold, *P*<0.05), BMP-2 (2.41-fold, *P*<0.05) and PTH treatment (2.33-fold, *P*<0.01), respectively. The expression returned to basic level at 24 h after treatment, except for treatment with BMP-2 (1.69-fold, *P*<0.05) and PTH (1.76-fold, *P*<0.05) (Figure 2d). For the subcellular localization of Arl6ip5 protein in osteoblasts, we observed that both exogenous Arl6ip5-EGFP protein and endogenous Arl6ip5 was highly overlapped with the ER as traced by ER-tracker and protein calnexin in POBs (Figure 2e) and in UAMS-32 cells (data not shown). In addition, endogenous Arl6ip5 was not immunolocalized to Golgi body as shown by the coimmunostaining with the Golgi protein GM130 (data not shown).

### Arl6ip5 regulates osteoblast proliferation and differentiation

**Figure 3:**
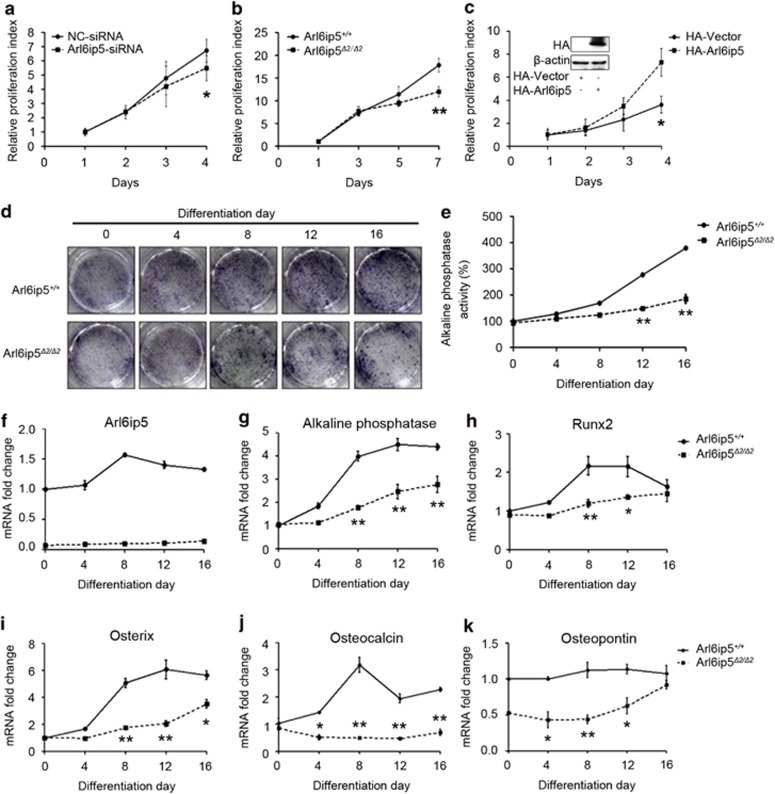
Arl6ip5 affects osteoblast proliferation and differentiation. Cell proliferation in UAMS-32 cells with Arl6ip5-siRNA (**a**) and HA-tagged Arl6ip5 (**c**) treatments were analyzed with MTT assay. The proliferation rate between Arl6ip5^*+/+*^ and Arl6ip5^*Δ2/Δ2*^ POBs (**b**) was also compared. Arl6ip5^*+/+*^ and Arl6ip5^*Δ2/Δ2*^ POBs were cultured in differentiated medium and evaluated for ALP staining (**d**), ALP activity (**e**) and mRNA levels of *Arl6ip5*, *ALP*, *Runx2*, *osterix*, *osteocalcin* and *osteopontin* (**f**–**k**). *P<0.05; **P<0.01 by *Student*'s *t*-test. In all panels except **d**, *n*=3. In **d**, representative results were shown from three independent experiments

To further understand the possible function of Arl6ip5 in osteoblast, a verified small interfering RNA (siRNA) (Supplementary Figure S5) was used to knock down Arl6ip5 expression in UAMS-32 cells. The 3-(4,5-dimethylthiazol-2-yl)-2,5-diphenyltetrazolium bromide (MTT) assay displayed that silence of Arl6ip5 reduced cell proliferation (Figure 3a), which was also observed in Arl6ip5^*Δ2/Δ2*^ POBs when compared with Arl6ip5^*+/+*^ POBs (Figure 3b). On the contrary, overexpression of Arl6ip5 in UAMS-32 cells with HA-tagged mouse Arl6ip5 (HA-Arl6ip5) significantly increased cell proliferation (Figure 3c). For osteoblast differentiation, the ALP-positive cells and the ALP activity in cultured Arl6ip5^*+/+*^ POBs were increased in time-dependent manner, but were just slightly changed in cultured Arl6ip5^*Δ2/Δ2*^ POBs (Figures 3d and e). The expression of osteoblastic differentiation markers, *ALP*, *Runx2*, *osterix*, *osteocalcin* and *osteopontin* in Arl6ip5^*Δ2/Δ2*^ POBs were also relatively lower compared with Arl6ip5^*+/+*^ POBs (Figures 3f–k).

### Arl6ip5 regulates ER calcium and activated CaM pathway

**Figure 4:**
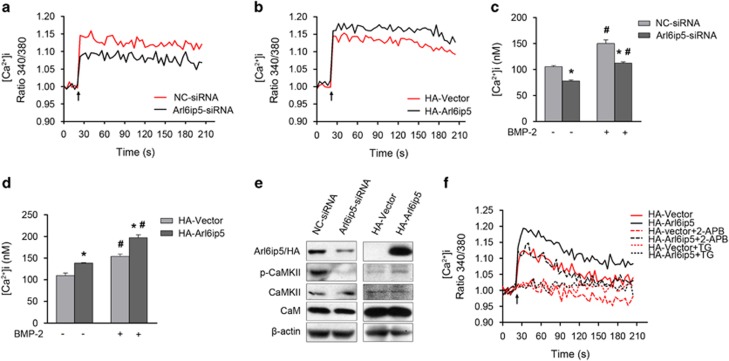
Arl6ip5 regulates intracellular calcium level and Ca^2+^-CaM signaling. ATP-stimulated [Ca^2+^]_i_ in a time course was analyzed in UAMS-32 cells received Arl6ip5-siRNA (black) and its negative control (NC-siRNA) (red) for 72 h (**a**) and in UAMS-32 cells stably transfected with pcDNA3.1(+) (HA-vector) (red) or HA-Arl6ip5 (black) (**b**). In **a** and **b**, the data shown are representative of at least four different experiments. (**c** and **d**) [Ca^2+^]_i_ levels in BMP-2-treated UAMS-32 cells. Arl6ip5-siRNA and NC-siRNA treated (72 h) or HA-Vector and HA-Arl6ip5 stably expressed UAMS-32 cells were cultured with BMP-2 (100 ng/ml) for 6 h. *HA-Arl6ip5 or Arl6ip5-siRNA *versus* control, *P*<0.05; ^#^BMP-2 treatment *versus* no treatment, *P*<0.05. Error bars represent the mean±S.E.M., *n*=3. (**e**) Arl6ip5 activates the Ca^2+^-CaM signaling pathways. In UAMS-32 cells with HA-Arl6ip5 stably overexpressed or Arl6ip5-siRNA transiently transfected, the levels of the signal molecules were analyzed by western blotting. (**f**) UAMS-32 cells stably transfected with HA-Vector or HA-Arl6ip5 were analyzed for ATP-stimulated [Ca^2+^]_i_. 2-APB (100 *μ*M) and TG (1 *μ*M) were added to the cell culture for 30 min before ATP stimulation. Representative results were shown from three independent experiments. In panel **a**, **b** and **f**, arrows indicated ATP stimulation

The homeostasis of intracellular Ca^2+^ level ([Ca^2+^]_i_), which could be modulated by some ER localized proteins, is important for osteoblast differentiation.^4,22^ Arl6ip5 was an ER-resident protein in osteoblast and could be evoked by Ca^2+^ deletion,^19^ therefore, we evaluated whether this protein was also involved in the regulation for [Ca^2+^]_i_ in osteoblasts. Our results indicated that ATP stimulated [Ca^2+^]_i_ were decreased in Arl6ip5 knocked-down cells and in Arl6ip5^*Δ2/Δ2*^ POBs (Figure 4a and Supplementary Figure S6) but increased in Arl6ip5-overexpressed UAMS-32 cells (Figure 4b) compared with respective controls. Moreover, in BMP-2-treated UAMS-32 cells, silence of Arl6ip5 decreased but overexpression of Arl6ip5 increased [Ca^2+^]_i_ level (Figures 4c and d). By measuring the cytosolic Ca^2+^ peak,^23^ we found that Arl6ip5 deficiency decreased ER Ca^2+^ store in POBs and Arl6ip5 stable overexpression led to a significant increase in ER Ca^2+^ levels in UAMS-32 cells (Supplementary Figure S7).

Intracellular Ca^2+^ activates and affects many signaling pathways that modulate cell differentiation, such as CaM-CaMKII-NFATc1 pathway.^4^ Despite the CaM protein level was not changed, the phosphorylated CaMKII was decreased in Arl6ip5 knocked-down cells and increased in Arl6ip5 overexpressed cells (Figure 4e). IP3Rs rather than RyRs were expressed in UAMS-32 cells (data not shown). To further analyze the activation mechanism of Arl6ip5 on Ca^2+^ channel, treated cells with 2-aminoethoxydiphenyl borate (2-APB), the inhibitor of IP3Rs^24^ completely blocked Ca^2+^ release from the IP3R channel in control cells but partially reduced the [Ca^2+^]_i_ levels in Arl6ip5 stable overexpressed UAMS-32 cells (Figure 4f). However, thapsigargin (TG), an ATPase inhibitor for SERCA,^25^ completely inhibited Ca^2+^ release from the ER in both controls and Arl6ip5 stable overexpressed cells (Figure 4e).

### Arl6ip5 deficiency activates ER stress-mediated apoptosis in osteoblast

**Figure 5:**
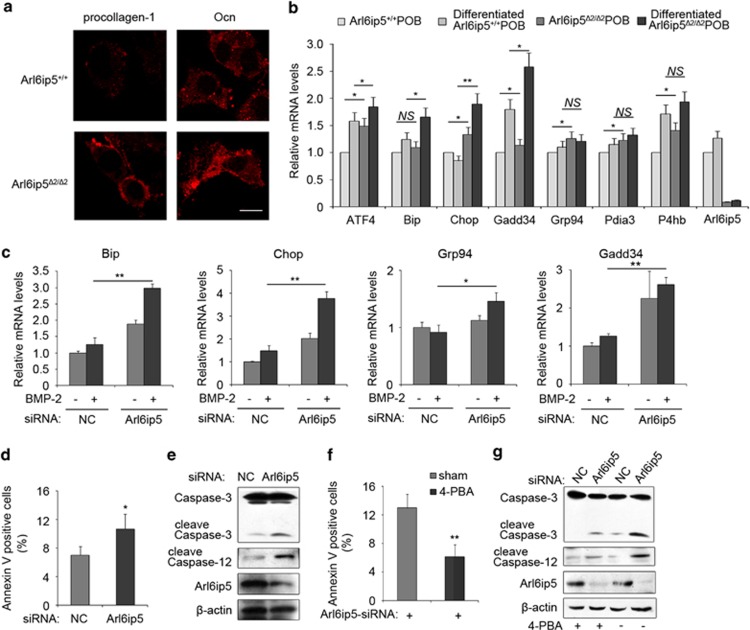
Deficiency of Arl6ip5 in osteoblasts induced ER stress and apoptosis. (**a**) Immunofluorescence staining using antibodies for procollagen-1 and osteocalcin (Ocn) in Arl6ip5^+/+^ and Arl6ip5^*Δ2/Δ2*^ POBs. Scale bar, 10 *μ*m. (**b**) Q-PCR analysis was used to detect the mRNA level for ER stress-related genes in POBs of Arl6ip5^+/+^ and Arl6ip5^*Δ2/Δ2*^ without or with differentiated medium treatment. *n*=3. (**c**) UAMS-32 cells first received NC-siRNA or Arl6ip5-siRNA treatment for 48 h, then received BMP-2 (100 ng/ml) for 24 h. Q-PCR was used to detect the mRNA level for ER stress-related genes. *n*=3. (**d** and **e**) The UAMS-32 cells received NC-siRNA or Arl6ip5-siRNA treatment together without (**d** and **e**) or with 4-PBA (10 mM) (**f** and **g**) were stained with Annexin-V and the proportion of Annexin-V-positive cells were calculated (**d** and **f**, *n*=4) or used for immunoblotting for the expression of caspase-3, caspase-12, Arl6ip5 and *β*-actin (**e** and **g**). For the immunoblotting, representative results were shown from three independent experiments. In all panels, error bars represent the mean±S.E.M. **P<0.01, *P<0.05 based on ANOVA in **b** and **c** and *Student*'s *t-*test in **d** and **f**

The disturbance of ER calcium homeostasis leads to accumulation of unfolded proteins in ER, thereby initiating ER stress and activating UPR.^11^ Immunostaining showed that the bone matrix proteins, such as procollagen-1 and osteocalcin (Ocn), were accumulated in ER of Arl6ip5^*Δ2/Δ2*^ POBs but not in Arl6ip5^+/+^ POBs (Figure 5a). UPR target genes P4hb, Grp94 and Pdia3, which help in relieving ER stress, and the genes Chop and Gadd34, which mediate pro-apoptosis signal of ER stress,^12^ were significantly increased in Arl6ip5^*Δ2/Δ2*^ POBs compared with Arl6ip5^+/+^ (Figure 5b). Moreover, the mRNA levels of Chop and Gadd34 were also increased significantly in differentiated Arl6ip5^*Δ2/Δ2*^ POBs compared with Arl6ip5^+/+^ POBs (Figure 5b). However, the XBP1s (spliced form of XBP1) was not detected, even in differentiated Arl6ip5^*Δ2/Δ2*^ POBs and Arl6ip5^+/+^ POBs (data not shown). The expression of Chop and Gadd34 genes were also increased in the tibia extracts from Arl6ip5^*Δ2/Δ2*^ mice (Supplementary Figure S8) and in Arl6ip5 knocked-down UAMS-32 cells (Supplementary Figure S9). BMP-2 treatment slightly enhanced the expression of Bip, Chop and Gadd34 in UAMS-32 cells, but the enhancement was obviously raised after Arl6ip5 was silenced (Figure 5c).

As Chop and Gadd34 mediates the pro-apoptosis signal of UPR.^12,16^ The elevated Chop and Gadd34 expression suggested that apoptosis may be initiated by Arl6ip5 deficiency in osteoblasts. As we suspected, the apoptotic osteoblasts was significantly increased and mRNA level of anti-apoptosis protein Bcl-2 was decreased in Arl6ip5^*Δ2/Δ2*^ mice tibia when compared with their controls (Supplementary Figure S10). In consistence, the Annexin-V-positive cells (Figure 5d) and the level of cleaved caspase-3 and caspase-12 proteins (Figure 5e) were markedly increased in Arl6ip5 knocked-down UAMS-32 cells. The pro-apoptotic genes Bim-1 and Puma, but not anti-apoptotic gene Bcl-2, were also raised after silence of Arl6ip5 in UAMS-32 cells (Supplementary Figure S11). Moreover, Annexin-V-positive cells (Figure 5f) and cleaved caspase-3 and caspase-12 proteins (Figure 5g) were markedly decreased in Arl6ip5 knocked-down cells by treatment with 4-phenylbutyrate acid (4-PBA), a chemical chaperon that prevents protein aggregation and relieves ER stress.

**Figure 6:**
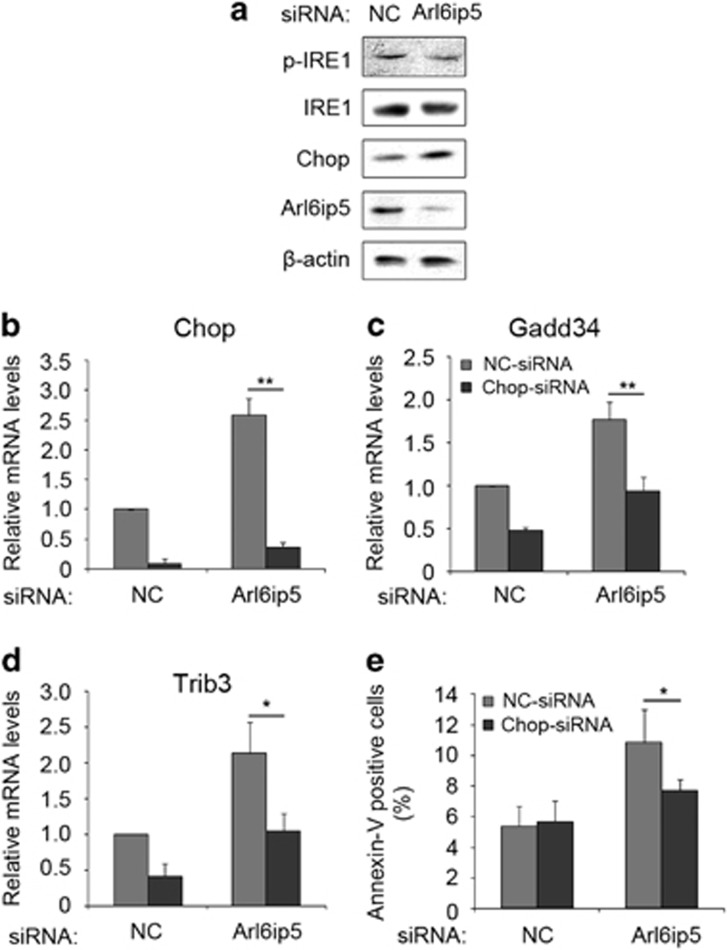
Apoptosis mediated by Arl6ip5 interference is regulated by Chop. (**a**) Immunoblotting was used to detect the protein level for p-IRE1*α*, IRE1, Chop, Arl6ip5 and *β*-actin in the UAMS-32 cell with NC-siRNA or Arl6ip5-siRNA treatment for 72 h. Representative results were shown from three independent experiments. (**b**–**e**) UAMS-32 cells first received NC-siRNA or Chop-siRNA for 24 h, then the medium was changed and further received NC-siRNA or Arl6ip5-siRNA for 72 h. Q-PCR was used to analyze the mRNA levels of Chop (**b**), GADD34 (**c**) and Trib3 (**d**). The apoptotic cells were stained with Annexin-V and the proportion were calculated (**e**). *n*=4. In all panels, error bars represent the mean±S.E.M. **P<0.01, *P<0.05 based on ANOVA

IRE1 and Chop involves in the ER stress-mediated apoptosis.^16^ Although the activity of IRE1 was not changed (Figure 6a), the protein expression of Chop was markedly increased in Arl6ip5 knocked-down cells compared with controls (Figure 6a). The mRNA levels of Trib3, one downstream target gene of Chop,^26^ was also increased (Supplementary Figure S12). Silence of Chop by siRNA (Figure 6b), apparently decreased the expression of Gadd34 and Trib3 (Figures 6c and d), and the number of Annexin-V-positive cells (Figure 6e) in Arl6ip5 knocked-down cells.

### The regulation of Arl6ip5 on osteoblast differentiation and proliferation needs Chop

**Figure 7:**
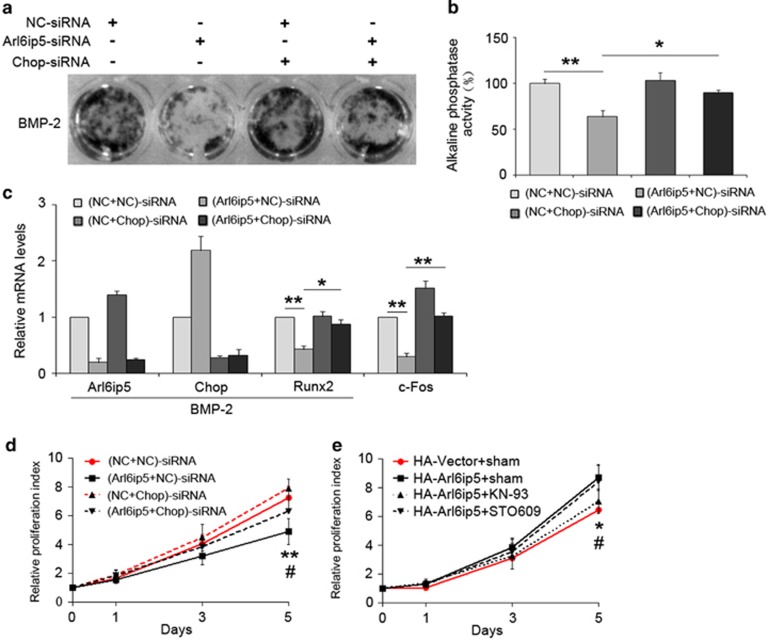
Chop involves in the regulation of Arl6ip5 on osteoblast proliferation and differentiation. (**a**–**c**) Double siRNA-treated UAMS-32 cells received with or without BMP-2 (100 nM) treatment for 5 days to induced differentiation, then evaluated by ALP staining (**a**) and ALP activity analysis (**b**) and mRNA levels of Arl6ip5, Chop, Runx2 (**c**, with BMP-2 treatment) and c-Fos (**c**, without BMP-2 treatment). For the panels of ALP activity analysis and mRNA detection, **P<0.01, *P<0.05 based on ANOVA. (**d**) Cell proliferation in UAMS-32 cells with double siRNA treatment were analyzed with MTT assay. **(NC+NC)-siRNA *versus* (NC+Arl6ip5)-siRNA, *P*<0.01; ^#^(Chop+Arl6ip5)-siRNA *versus* (NC+Arl6ip5)-siRNA, *P*<0.05. (**e**) Cell proliferation in HA-Arl6ip5 overexpressed UAMS-32 cells with or without KN-93 (1*μ*M) and STO-609 (1*μ*M) treatment were analyzed with MTT assay. *HA-Vector+sham *versus* HA-Arl6ip5+sham, *P*<0.05; ^#^HA-Arl6ip5+KN-93 *versus* HA-Arl6ip5+sham, *P*<0.05. In all panels, *n*=4. Error bars represent the mean±S.E.M.

Chop also involves in the regulation of osteoblast differentiation, and the proliferation in some cells.^27, 28, 29^ In this study, knocking down the Chop significantly increased the ALP-positive cells and ALP activity (Figures 7a and b) and the expression of Runx2 in Arl6ip5-siRNA-treated UAMS-32 cells (Figure 7c). For osteoblast proliferation, treating the cells with Chop-siRNA significantly enhanced the expression of c-Fos (Figure 7c), one key transcription factor for cell proliferation, and improved the proliferation ability of Arl6ip5 knocked-down UAMS-32 cells (Figure 7d). Moreover, the proliferation ability of Arl6ip5 deficiency UAMS-32 cells was significantly rescued by the treatment with CaCl_2_ (Supplementary Figure S13A). The proliferation ability (Figure 7e) and c-Fos mRNA level of HA-Arl6ip5 overexpressed cells (Supplementary Figure S13B) was inhibited by CaMK kinase inhibitors KN-93 but not STO609 treatments.

### Osteoblastic Arl6ip5 regulates RANKL expression

**Figure 8:**
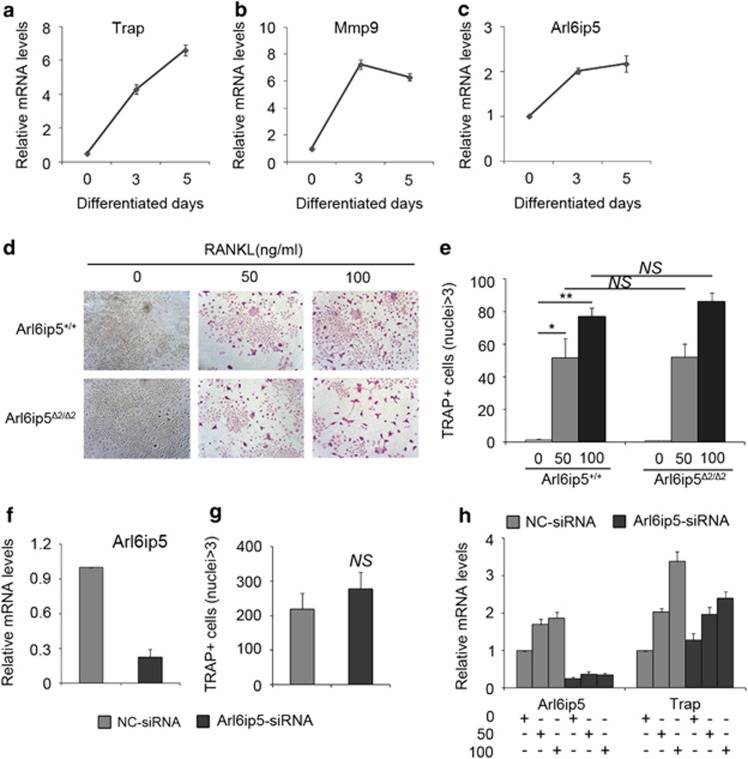
Arl6ip5 interference does not affect osteoclastogenesis. (**a**–**c**) BMMs were differentiated in the presence of 10 ng/ml M-CSF and 100 ng/ml RANKL for indicated days, then mRNA of Trap (**a**), Mmp9 (**b**) and Arl6ip5 (**c**) was detected, *n*=3. (**d** and **e**) BMMs from Arl6ip5^+/+^ and Arl6ip5^*Δ2/Δ2*^ mice were differentiated with 10 ng/ml M-CSF and 50 or 100 ng/ml RANKL for 6 days in 24 wells, then for TRAP staining. Representative fields were shown. Scale bars=100 *μ*m (**d**). Histograms quantify the average numbers of multinucleated TRAP+ cells. Data represent mean±S.E.M. derived from three independent experiments, each using BMMs from three littermates (**e**). (**f** and **g**) Raw264.7 cells received NC-siRNA or Arl6ip5siRNA were differentiated in the presence of 50 or 100 ng/ml RANKL for 5 days, then stained for TRAP activity and detected for the mRNA expression of Arl6ip5 and Trap. Histograms quantify the average numbers of multinucleated TRAP+ cells (**f**). Q-PCR verified the downregulation of Arl6ip5 (**g**). Q-PCR analyzed the mRNA expression of Arl6ip5 and Trap (**h**). *n*=3. Data represent mean±S.E.M. NS, nonsignificant. *P<0.05; **P<0.01

The increase of osteoclast formation in Arl6ip5^*Δ2/Δ2*^ mice implicated the role of Arl6ip5 in osteoclastogenesis. As markers Trap and Mmp9, Arl6ip5 expression was increased during osteoclast differentiation (Figures 8a–c), however, the TRAP-positive osteoclast formation was comparable between BMMs from wild-type mice and from Arl6ip5^*Δ2/Δ2*^ mice (Figures 8d and e). In consistence, silenced Arl6ip5 expression with siRNA in Raw264.7 cells slightly but not significantly (P=0.18) increased the osteoclast formation when compared with negative control siRNA-treated cells (Figures 8f and g). There was also no difference in the expression level of Trap (Figure 8h).

**Figure 9:**
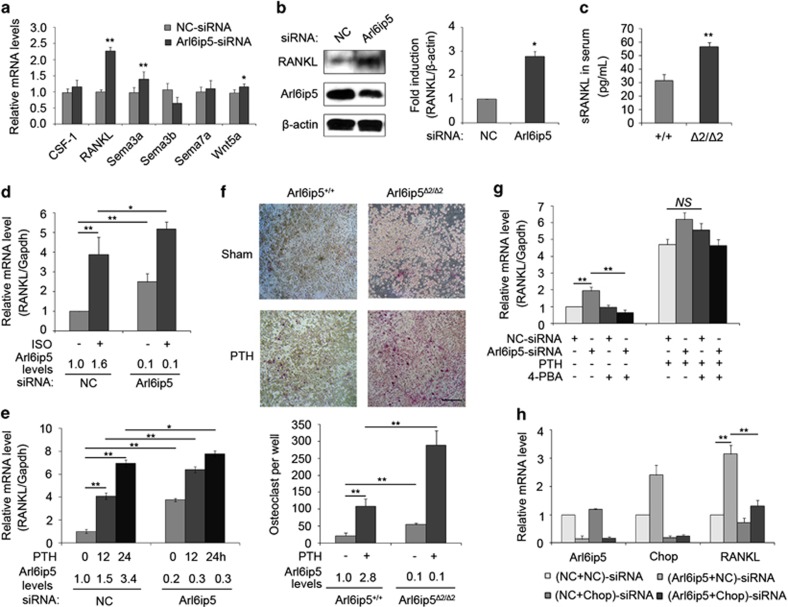
Osteoblastic Arl6ip5 deficiency enhances osteoclastogenesis via increasing RANKL expression. (**a**) Q-PCR was used to detect the expression of osteoblast–osteoclast coupling factors. *n*=6. Data represent mean±S.E.M. *P<0.05 and **P<0.01 by *Student*'s *t-*test. (**b**) Immunoblotting was used to measure the protein expression of RANKL, Arl6ip5 and *β*-actin in NC-siRNA and Arl6ip5-treated UAMS-32 cells. The densitometry results (arbitrary units) of three independent experiments were shown as a bar graph. Bar represents mean±S.E.M. *P<0.05 by *Student*'s *t-*test. (**c**) Serum was collected from 16-week-old Arl6ip5^*Δ2/Δ2*^ (*n*=6) and their wild-type littermates (Arl6ip5^+/+^) (*n*=6) and the sRANKL levels were analyzed with ELISA kit. Each bar represents mean±S.E.M. *P<0.05, *Student*'s *t-*test. (**d** and **e**) Q-PCR analyzed the RANKL transcription induced by ISO (**d**) and PTH (**e**) treatment in UAMS-32 cells with Arl6ip5-siRNA treatment. The mRNA level of Arl6ip5 was shown under the panel. (**f**) Osteoclast formation in co-cultures of Arl6ip5^+/+^, Arl6ip5^*Δ2/Δ2*^ POBs and bone marrow macrophages (BMMs) as osteoclast (OC) precursors, treated with or without PTH (100 nM). Representative images were shown in the upper panel. Scale bar=1 mm. Histograms quantify the average numbers of multinucleated TRAP+ cells. (**g**) UAMS-32 cells were treated with NC-siRNA and Arl6ip5-siRNA for 48 h, then received PTH (100 nM) with or without 4-PBA (10 mM) for 24 h. The transcription of RANKL was analyzed by Q-PCR. (**h**) UAMS-32 cells first received NC-siRNA or Chop-siRNA for 24 h, then the medium was changed and further received NC-siRNA or Arl6ip5-siRNA for 72 h. Q-PCR was used to analyze the mRNA levels of Chop, Arl6ip5 and RANKL. For **d**–**h**, bar represents mean±S.E.M. (*n*=3). *P<0.05 and **P<0.01 by ANOVA

As osteoblast lineage cells (e.g., osteoblast and osteocyte) are coupling with osteoclast and regulate osteoclastogenesis,^30^ it is possible that the increased of osteoclast number is caused by the aberrant expression of coupling factors in Arl6ip5 deficiency osteoblast. To verify this hypothesis, the well-demonstrated coupling factors (CSF-1, RANKL, Sema3a, Sema3b, Sema7a and Wnt5a) that express in osteoblast and regulate osteoclastogenesis were screened for their expression in Arl6ip5 knocked-down UAMS-32 cells. We found that the basic RANKL mRNA was significantly increased (2.3-fold, *P*<0.05) in Arl6ip5-siRNA-treated UAMS-32 cells (Figure 9a). In consistence, the protein level of RANKL in Arl6ip5-siRNA-treated UAMS-32 cells was increased nearly threefold compared with control siRNA-treated cells (Figure 9b). The RANKL mRNA level in tibia extracts from Arl6ip5^*Δ2/Δ2*^ mice was also significantly higher than in wild-type mice (Figure 1e). Moreover, soluble RANKL (sRNAKL) in serum from Arl6ip5^*Δ2/Δ2*^ mice was nearly twofold higher compared with Arl6ip5^+/+^ mice (Figure 9c). Furthermore, the increase of RANKL expression by treatment with PTH, isoproterenol hydrochloride (ISO) or 1,25-(OH)_2_D_3_ was further increased in Arl6ip5 knocked-down osteoblast (Figures 9d and e and Supplementary Figure S14). The basic and PTH-induced sRANKL level in the culture medium of Arl6ip5-siRNA-treated cells or Arl6ip5 KO POBs were also higher compared with the culture medium of control cells (Supplementary Figure S15). RANKL is essential for osteoclastogenesis.^1^ The numbers of TRAP-positive osteoclast (Figure 9f) and pit formation (Supplementary Figure S16) were much more in the co-culture containing Arl6ip5^*Δ2/Δ2*^ osteoblast than the co-culture containing Arl6ip5^+/+^ osteoblast. To further investigate the mechanism for the regulation of Arl6ip5 on RANKL expression, H-89, an inhibitor of PKA activity and 4-PBA, the chemical chaperon as indicated above were used. The induced RANKL expression in Arl6ip5 knocked-down UAMS-32 cells was lower to normal with 4-PBA but not with H-89 treatment (Figure 9g and Supplementary Figure S17), however, 4-PBA treatment had no effect on the RANKL expression induced by PTH (Figure 9g). The expression of basic RANKL as Arl6ip5 deficiency was further increased after cells treated with ATF4-siRNA (Supplementary Figure S18), but significantly decreased after Chop gene was interfered (Figure 9h).

## Discussion

The study with gene KO mice is important to unravel the *in vivo* functions of one gene. The deletion of exon2 of Arl6ip5 mediated by Cre-LoxP strategy resulted in a null allele with Arl6ip5 loss-of-function in tissues from homozygous mutant mice. The phenotype analysis of Arl6ip5 KO mice revealed an unrecognized role of Arl6ip5 in bone metabolism. Specifically, gene ablation of Arl6ip5 in mice decreased bone mass owing to diminution of osteoblast differentiation and increase of osteoclast formation. Mechanistically, deficiency of Arl6ip5 appears to inhibit osteoblast proliferation and differentiation via the disturbance of calcium homeostasis and induction of apoptosis that mediated by ER stress, moreover, favor osteoclastogenesis through enhancing RANKL expression in osteoblast.

Arl6ip5, initially cloned from human tracheal bronchial epithelial cells after treatment with all-trans retinoic acid (ATRA), was regarded as a novel cell differentiation-associated gene, and regulated by differentiation inducers such as ATRA, phorbol-12-myristate-13-acetate, arabinoside and hemin.^17,31,32^ In human myeloid leukemia cells, Arl6ip5 was associated with ATRA-induced cellular differentiation and cellular proliferation.^32^ In neurite growth, GTRAP3-18, the rat homologue of Arl6ip5, was reported to be a negative regulator for neuronal differentiation.^33^ In this study, the expression of Arl6ip5 was increased during osteoblast differentiation. Moreover, Arl6ip5 mRNA level was induced by osteotropic factors, such as PTH, TGF-*β*1, BMP-2 and Dex in UAMS-32 cells. Immunostaining of Arl6ip5 demonstrated that Arl6ip5 is an ER localized protein in osteoblast. These findings suggested that Arl6ip5 may be related to osteoblast proliferation and differentiation, which was supported by the findings that Arl6ip5 deficiency *in vitro* impaired osteoblast proliferation and differentiation.

For the mechanisms underlying, we observed that Arl6ip5 regulated intracellular calcium level via its regulation on ER calcium channel that mediates calcium output from ER. It is important to note that the potential channels that is regulated by Arl6ip5 are different from the IP3R channel, as the IP3Rs inhibitor 2-APB only partially inhibited the increase of Ca^2+^ that is mediated by Arl6ip5 was overexpressed. Calcium signaling has been well demonstrated for its role in osteoblast proliferation and differentiation owing to its regulation on the expression and activity of the transcription factors, which are important for osteoblast differentiation, maturation and bone formation, for example, c-fos, Runx2 and osterix.^10,34,35^ It is plausible that the decrease of Runx2 and osterix was subsequent to the impaired calcium signaling in Arl6ip5 deficiency osteoblasts, although the activity of these transcription factors was not observed in this study. However, a recent study showed that mice with deficiency of CAMKK2, one upstream kinase of CaMKI and CaMKIV, possessed higher trabecular bone mass in their long bones and more OBs and fewer multinuclear OCs.^36^ Whereas the activity of CaMKI and CaMKIV was not detected in this study, the activity of CaMKII, which is ubiquitously expressed and well studied in osteoblast differentiation,^4,10,37^ was decreased upon Arl6ip5 interference. As the activation of CaMKII by Ca^2+^/CaM binding and its autophosphorylation are CaMKK independent,^38^ it is difficult to evaluate the role of CaMKKs in the impairment of osteoblast differentiation owing to Arl6ip5 deficiency. However, for osteoblast proliferation, studies with inhibitor KN-93 (generous to CaMKI, CaMKIV and CaMKII) and STO-609 (specific for CaMKK and the downstream targets CaMKI and CaMKIV) showed that CaM-CaMKII but not CaMKK was involved for the regulation of Arl6ip5 on osteoblast proliferation. Further study with specific shRNA or siRNA for CaMKKs and CaMKs are warranted.

We also observed that Arl6ip5 deficiency induced ER stress, as indicated by the increase of ER stress-related genes. Mild ER stress is helpful for restoring cellular homeostasis, however, the persistent and unalleviated ER stress elicits apoptosis.^12,16^ In this study, the significant increase of Chop and its target gene Gadd34, two representative genes for the regulation of ER stress-mediated apoptosis, indicated the UPR to ER stress shift from pro-survival to pro-apoptosis in the cells with Arl6ip5 deficiency. The apoptosis was demonstrated by the *in vivo* and *in vitro* evidence that TUNEL-positive or Annexin-V-positive cells and the protein expression of cleave caspase-3 was increased in Arl6ip5 deficiency cells. Moreover, the increased processing of caspase-12, a well-demonstrated indicator for ER stress-induced apoptosis,^39^ and the reversed effect of chemical chaperon 4-PBA suggested that the apoptosis upon Arl6ip5 knocked-down is dependent on the ER stress.

IRE1- and Chop-mediated apoptosis pathway has a central role in ER stress-mediated apoptosis.^16^ In Arl6ip5 knocked-down osteoblast, the mRNA and protein levels of Chop were increased. However, the activity of IRE1 was comparable between Arl6ip5 knocked-down and normal expressed cells. Moreover, knocked-down Chop with specific siRNA reversed the expression of its target genes and the apoptosis phenotype in Arl6ip5 knocked-down cells. These findings supported that Chop was involved in the regulation of apoptosis that mediated by Arl6ip5 interference. Chop regulates the expression of genes that related with apoptosis,^40^ such as Ero1, Bcl-2 and Trib3. We found that Trib3 was markedly increased but Ero1 and Bcl-2 expression was not changed in Arl6ip5 knocked-down cells. This differential may be cell type or stimulus dependent. Further study with gene expression array would be helpful for revealing the underlying mechanisms. Chop was shown to inhibit the differentiation of committed osteoblasts such as POBs but promote differentiation of ST-2, a multipotential mesenchymal progenitor cell line.^27,28^ In this study, knocked-down Chop increased the differention and proliferation of Arl6ip5 deficiency UAMS-32 cells possibly via its regulation on the expression of Runx2 and c-Fos. Our results support the findings that Chop inhibits differentiation in committed osteoblast, as revealed by Shirakawa *et al.*^28^ UAMS-32 cells was isolated from mouse bone marrow cells and exhibited phenotypic characteristics of stromal/osteoblastic cells,^41^ but whether this cell line has multipotential property or just a committed osteoblst is still not clear. Further studies with Chop-specific deficiency in osteoblast on different stages (osteoblast progenitors, stromal marrow cells, pre-osteoblast and mature osteoblast) is important for the dual roles of Chop in osteoblast differentiation.

The *in vivo* study with Arl6ip5 KO mice indicated that the osteoclastogenesis was enhanced. However, the *in vitro* study with Arl6ip5 deficiency BMMs and Arl6ip5 knocked-down Raw264.7 cells showed that Arl6ip5 impaired in osteoclast progenitors had no effect on osteoclast formation, which prompted us to test the hypothesis that Arl6ip5 in osteoblasts have a critical role in osteoclastogenesis. We found that osteoblastic Arl6ip5 insufficiency significantly increased the expression of RANKL, the key factor for osteoclastogenesis. The subsequent *in vitro* study revealed the basal and stimuli (PTH, ISO and VitD3) induced RANKL gene expression and sRNAKL level were also significantly increased in Arl6ip5 knockdown osteoblast and Arl6ip5 KO POBs, moreover, the co-culture assay containing Arl6ip5^*Δ2/Δ2*^ POBs with osteoclast precursors found more osteoclast formation and pit formation. These findings suggested that osteoblastic Arl6ip5 deficiency not only impaired osteoblast differentiation but also provided a microenvironment that suitable for osteoclastogenesis.

PTH and isoproterenol regulate RANKL expression in osteoblast via PKA-CREB and PKA-ATF4 pathway, respectively.^42, 43, 44^ However, it was the ER stress-related signaling but not the PKA-dependent pathway involved in the induction of basic RANKL transcription in Arl6ip5 knocked-down cells, as 4-PBA treatment but not H-89 treatment blocked the effect of Arl6ip5 knocked-down on RANKL expression. The further study found that Chop, rather than ATF4, was the key factor to mediate the induction of basic RANKL expression in Arl6ip5 knocked-down cells. ATF4 has been demonstrated for its regulation on RANKL expression with ATF4 deficiency POBs,^43^ and ATF4 also regulates the genes that are important for alleviating ER stress.^12^ However, ATF4 interference by siRNA in this study, did not reduced the basic RANKL expression induced by ER stress as Arl6ip5 deficiency, indicated that ER stress mediates RANKL expression via ATF4-independent mechanisms. During ER stress, Chop also regulates the genes that involved in apoptosis and protein synthesis.^40,45^ It is still not clear whether the regulation mechanisms for these genes also implicated in the regulation of Chop on RANKL expression. Interestingly, the transgenic mice with Chop overexpressing showed impaired osteoblastic function and osteopenia owing to increased osteoblast apoptosis.^46^ Although further studies about the detailed mechanisms in the regulation of RANKL transcription are warranted, it is possible that the osteoclastogenic effect mediated by Chop found in this study also contributes to the osteopenia phenotype for Chop-overexpressing mice.

In conclusion, our results revealed a novel effect of osteoblastic Arl6ip5 in bone formation and signal couplings between osteoblast and osteoclast via its regulation on cellular homeostasis in bone metabolism. Arl6ip5 is an ER-resident protein and regulated by bone metabolism factors in osteoblast. Deficiency of Arl6ip5 in osteoblast disturbs the calcium homeostasis and induces ER stress-mediated apoptosis, then impairs osteoblast proliferation and differentiation, moreover, produces an osteoclastogenic microenvironment by the induction of RANKL. Therefore, the loss of Arl6ip5 disturbs the homeostasis of osteoblast and skeleton. However, the use of mice with Arl6ip5 global deficiency compromised the findings. Current efforts are focus on the cell lineage-specific conditional inactivation of the Arl6ip5 allele and the identification of Arl6ip5-interacting proteins in an attempt to gain further insights into the mechanism of activity and function of Arl6ip5 in skeletal disease.

## Materials and Methods

### Animals

Arl6ip5 KO mice (Arl6ip5^*Δ2/Δ2*^) were constructed as described before^21^ and kept in a pathogen-free environment in a standard breeding room. Mice used in this study were intercrossed for at least 10 generations and were maintained in a C57BL/6J background. Wild-type (Arl6ip5^*+/+*^) littermates were used as controls. Mouse experiments were approved by the Jiangsu Institute of Nuclear Medicine Animal Ethical Committee and conducted in accordance with Animal Care and Use Committee of the Model Animal Research Centre.

### Reagents and antibodies

Fetal bovine serum (FBS), L-glutamine, antibiotics, alpha-modified essential medium (*α*-MEM) and trypsin/EDTA were obtained from Gibco (Life Technologies, Grand Island, NY, USA). PTH, Dex, 1,25-(OH)_2_D_3_, MTT, DMSO, ascorbic acid, *β*-glycerophosphate, indomethacin, TG, 2-APB, 4-PBA, KN-93 and STO-609 were purchased from Sigma (Sigma-Aldrich, St. Louis, MO, USA). ISO was from TCI (Tokyo Chemical Industry, Tokyo, Japan). TGF*β*1, BMP-2 and RANKL were from R&D (R&D Systems, Inc., Minneapolis, MN, USA). Type 2 collagenase was obtained from Worthington (Worthington Biochemical Corporation, Lakewood, NJ, USA). The antibodies for CaM, p-CaMKII and CaMKII were from Epitomics (Epitomics-an Abcam Company, Burlingame, CA, USA). Caspase-3 and caspase-12 antibodies from Cell Signaling (Cell Signaling Technology, Inc., Danvers, MA, USA). p-IRE1 (Ser724) and IRE1 antibodies were from Novus (Novus Biologicals, LLC, Littleton, CO, USA). Chop antibody from Beyotime (Beyotime Institute of Biotechnology, Nantong, China). Anti-HA and *β*-actin antibodies were from Santa Cruz (Santa Cruz Biotechnology, Inc., Santa Cruz, CA, USA). Arl6ip5 antibody was used as described before.

### Cells culture

UAMS-32 stromal/osteoblastic cell line was a gift from Professor Charles A O'Brien^41^ (University of Arkansas for Medical Sciences, Little Rock, AR, USA) and maintained in *α*-MEM containing 10% FBS, 2 mM L-glutamine and antibiotics. POBs were extracted from both genders of newborn neonatal mice calvaria with sequential type 2 collagenase digestion and cultured in *α*-MEM containing 15% FBS, 2 mM L-glutamine and antibiotics. The POBs used in this study were passaged 2–4 times. Raw264.7 cell were purchased from ATCC (American Type Culture Collection, Manassas, VA , USA) and maintained in *α*-MEM containing 10% FBS and antibiotics. For inducing POB differentiation, cells were cultured in medium containing 50 *μ*M ascorbic acid, 10 mM *β*-glycerophosphate, 100 *μ*M indomethacin and 100 nM Dex for the indicated time with regular medium changed. Non-adherent bone marrow cells were isolated from femurs and tibia of 60- to 90-day-old C57BL/6J mice and the suspended cells were collected after 48-h culture were used for co-culture assay. The UAMS-32 cells were treated with BMP-2 (100 ng/ml) and the Raw264.7 cells were treated with RANKL (100 ng/ml) to induce differentiation.

### Plasmids and siRNA

cDNA extracted from UAMS-32 cells was used. Full-length Arl6ip5 with HA tagged on C terminus (HA-Arl6ip5) was constructed with the primers (from 5′ to 3′): sense – CGCGGATCCGCCACCATGGACGTGAACCTCGCC; antisense – CGGTCTAGATTAAGCGTAGTCTGGGACGTCGTATGGGTACTCCCTCGCTTTGCTGATGTA and inserted into pcDNA-3.1(+) vector with *BamH*I and *Xba*I. Full-length Arl6ip5 with EGFP tagged on C terminus (Arl6ip5-EGFP ) was constructed with the primers (from 5′ to 3′): sense – GCGGAATTCATGGACGTGAACCTCG; antisense – GGTGGATCCCTCCCTCGCTTTGCTG and inserted into EGFP-N1 vector with *Eco*RI and *Bam*HI. The plasmids were verified by sequencing. Plasmids were transfected into UAMS-32 cells or POB with Lipofectamine 2000. UAMS-32 cells with stable plasmid expression were selected under 100 *μ*g/ml Geneticin (Sigma-Aldrich).

siRNA specific for Arl6ip5 and negative control siRNA were purchased from siGENOME SMART pool (Thermo Fisher Scientific, Rockford, IL, USA) and transfected using Lipofectamine 2000 (Life Technologies) according to the manufacturer's protocol. The sequence of siRNA is as below (5′ to 3′): UCUAUUACCAGACCAACAU. The siRNA for Chop and ATF4 was purchased from Ruibo (Guangzhou RiboBio Co., Ltd., Guangzhou, China). For double siRNA transfection, UAMS-32 cells first received NC-siRNA or Chop-siRNA or ATF4-siRNA for 24 h, then the medium was changed and further received NC-siRNA or Arl6ip5-siRNA for 72 h. The efficiency of siRNA treatments were evaluated by quantitative polymerase chain reaction (Q-PCR) or immunoblotting as indicated.

### Q-PCR analysis

The mRNA extracted from cells or tissues was first reverse transcribed to cDNA with High Capacity cDNA Reverse Transcription Kit (Applied Biosystems, Carlsbad, CA, USA), then analyzed on ABI Prism 7500 sequence detection system with SYBR Premix Ex Taq Mix (TaKaRa Bio Inc., Otsu, Japan) for the expression of *Arl6ip5*, *alkaline phosphatase*, *osteopontin, Trap, Ctsk, Mmp9, Bip, Chop, Grp94, Pdia3, P4hb, Gadd34, Trib3, c-Fos, β-actin* and *Gapdh*, the primers for these genes were retrieved from PrimerBank.^47^ For mRNA expression of *osterix* (Mm04209856_m1), *Runx2* (Mm00501580_m1), *osteocalcin* (Mm03413826_mH) and *RANKL* (Mm00441908_m1), the Taqman primers and probes indicated were used. All detections were in triplicate for each sample and data were normalized to *Gapdh* or *β-actin* levels (^*ΔΔ*^cT).

### Proliferation and apoptosis analysis

The cell proliferation was detected with MTT assay as previously indicated with some in-house modifications.^48^ Briefly, 1 × 10^3^ UAMS-32 cells with Arl6ip5 stable expression or 1 × 10^3^ UAMS-32 cells received the siRNA treatment for the indicated days or 2 × 10^3^ POBs were seeded on 96-well microplate, cultured with or without KN-93(1 *μ*M) and STO-609 (1 *μ*M) treatment for indicated days and then 2 mM MTT was added into the cultured medium in 1 : 10 (vol:vol) ratio and incubated in 37 °C for 2 h, then the medium was replaced with DMSO and the plate was continuously incubated in 37 °C for another half hour in the dark and the absorbance of colored solution at 470 nm was measured by SpetraMax M5 microplate reader (Molecular Devices, Sunnyvale, CA, USA). For the proliferation analysis of double-siRNA-treated UAMS-32 cells, 24 h after the second siRNA treatment, cells were trypsinized and seeded into 96-well microplate for MTT analysis. Apoptosis analysis with Annexin-V-FLUOS Staining Kit (Roche Applied Science, Indianapolis, IN, USA) and DeadEnd Colorimetric TUNEL System (Promega, Madison, WI, USA) followed the protocol provided.

### ALP staining and activity measurement

At first, 1 × 10^4^ POBs per well in 24-well culture plates were cultured and differentiated in *α*-MEM medium containing 50 *μ*M ascorbic acid, 10 mM *β*-glycerophosphate, 100 *μ*M indomethacin and 100 nM Dex for indicated time with regular medium changed. TRACP and ALP double-staining Kit (TaKaRa Bio Inc.) was used for ALP staining. Alkaline Phosphatase Assay Kit (Beyotime Institute of Biotechnology) was used for ALP activity analysis.

### Western blotting

For western blotting, total cytoplasmic protein was isolated with RIPA lysis buffer containing protease and phosphatase inhibitors (Halt Protease Inhibitor Cocktail) from Pierce (Thermo Fisher Scientific Inc.) and run on 7.5–12% SDS-PAGE gels. After blotting to PVDF membrane (Life Technologies), membranes were blocked for 60 min at room temperature and incubated overnight at 4 °C in buffer (TBS with 0.1% Tween 20 and 5% non-fat milk powder) containing diluted antibodies. Detection of primary antibodies was performed with HRP-conjugated secondary antibody. Immunoreactive bands were visualized with Chemiluminescent substrate (Thermo Fisher Scientific Inc.). Densitometric analysis was performed using Image J software (National Institutes of Health, Bethesda, MD, USA).

### ELISA and immunostaining

sRNAKL in culture medium or in serum was detected with mouse RANKL ELISA kit from R&D (R&D Systems, Inc.) following the manufacturer's protocol. C-telopeptide of Collagen alpha-1(II) chain (cTx-II) in mice serum was detected with ELISA Kit for Mouse C-telopeptide of Collagen alpha-1(II) chain from EIAab (Wuhan EIAab Science Co. Ltd., Wuhan, China). For immunofluorescence staining, cells with Arl6ip5-EGFP overexpressed were incubated with ER-tracker from Molecular Probe (Life Technologies) for 0.5 to 2 h as indicated by the protocols. For the endogenous Arl6ip5, cells were fixed in methanol and incubated with the PBST containing the diluted primary antibodies for Arl6ip5 and calnexin overnight. Secondary antibodies were goat anti-rabbit or anti-mouse antibody conjugated to Alexa Fluor 488 or 594 from Molecular Probe (Life Technologies). Images were acquired with Olympus Laser Scanning Confocal Microscope (Olympus Corporation, Tokyo, Japan). Image J software was used to merge images.

### [Ca^2+^]_i_ measurements

The [Ca^2+^]_i_ of UAMS-32 cells with stable expression of either HA-Arl6ip5 or control vector and cells transiently transfected with Arl6ip5-siRNA (Thermo Fisher Scientific Inc.) were measured by loading with 5 *μ*M Fura-2AM (Sigma-Aldrich) prepared in extracellular buffer with no calcium (125 mM NaCl, 5 mM KCl, 1.5 mM MgCl_2_, 20 mM HEPES, 10 mM glucose, pH7.4) for 30 min at 37 °C. For the stimulation, 200 *μ*M of ATP was added to the cells and the Ca^2+^ transients were recorded as the 340/380 nm ratio (R) of the resulting 510-nm emissions using SpetraMax plate reader (Molecular Devices). For inhibition experiments, cells were incubated for 30 min before analysis with one of the following inhibitors: 100 *μ*M 2-APB for blocking IP3Rs, 0.5 *μ*M TG for SERCA ER Ca^2+^ pump inactivation. The [Ca^2+^]_i_ levels were calculated as described previously using the equation [Ca^2+^]_i_=Kd *(R–R_min_)/(R_max_–R)(F380_max_/F380_min_),^49^ where R_min_ is the ratio at zero Ca^2+^, R_max_ is the ratio when Fura-2 is completely saturated with Ca^2+^, F380 _min_ is the fluorescence at 380 nm for zero Ca^2+^ and F380_max_ is the fluorescence at saturating Ca^2+^ and Kd=224 nM.

### Osteoclast formation assay and pit formation assay

Calvarial cells (POBs) isolated from neonatal Arl6ip5^*Δ2/Δ2*^ or wild-type mice were co-cultured with non-adherent bone marrow cells. POBs at a density of 1 × 10^3^ cells per well in 48-well plates were co-cultured with 2 × 10^4^ per well non-adherent bone marrow cells isolated from wild-type mice for 7–9 days in *α*-MEM containing 10% FBS and 100 nM PTH. One-half of the medium was replaced with fresh medium and PTH every 3 days. After 9 days co-cultures, cells were fixed and stained for TRAP using TRACP and ALP double-stain Kit (TaKaRa Bio Inc.). The osteoclast with three or more nucleus was calculated with Image J software. For resorption analysis, 1 × 10^4^ Raw264.7 cells were seeded into the 24-well culture plate that contained 2 × 10^3^ POBs and dentine slices (Immunodiagnostic Systems PLC, Boldon, UK), 100 nM PTH was added the next day and culture for 5–6 days with regular medium changed. Slices were incubated with 0.25 M ammonium hydroxide and sonicated for several times. The slices were stained with 0.1% toluidine blue in 0.5% sodium tetraborate for 5 min, washed with water and air dried before photographs were taken by reflected light microscope. The resorpted areas were measured by Image J software.

### Bone histomorphometric analysis

For bone histomorphometric analysis, 4-month-old Arl6ip5 KO mice (Arl6ip5^*Δ2/Δ2*^) of both genders and their wild-type littermates were used. *μ*CT and bone histomorphometry were carried out as previously described with slight modification.^50^ Briefly, for *μ*-CT analysis, tibia isolated from each mouse were scanned by using a cone-beam microfocus X-ray computed tomography (*μ*CT40; Scanco Medical AG, Brüttisellen, Switzerland), image acquisition was performed at 100 kV and 98 *μ*A with a 0.9-degree rotation between frames. The resolution of the *μ*CT images is 18.2 *μ*m. For dynamic bone histomorphometry, double calcein (10 *μ*g/g) (C-0875, Sigma, St. Louis, MO, USA) were intraperitoneally injected on 15 and 5 days mice (4-month-old Arl6ip5^*Δ2/Δ2*^ and their wild-type littermates) before they were killed. Then the tibias were harvested and embedded in methyl methacrylate. Serial sections were cut, viewed and imaged using fluorescence microscopy (Olympus IX53 microscope). The double calcein-labeled width of endosteum of cortical bone and trabecular bone was measured and the MAR was calculated as the interlabel width/labeling period. For static bone histomorphometry, tibias were fixed in 4% neutral buffered formalin, decalcified in EDTA (pH 7.2), embedded in paraffin and sectioned at a thickness of 5 *μ*m. The sections were stained with hematoxylin and eosin staining and TRAP staining and the number of osteoblast and osteoclast in the cortical bone of metaphysis were measured.

### Statistical analysis

Results are expressed as the mean±S.E.M. Statistical significance was identified by Student's *t*-test or ANOVA where appropriate, with probability *P*<0.05 being considered significant.

## Abbreviations

2-APB: 2-aminoethoxydiphenyl borate
4-PBA: 4-phenylbutyric acid
ALP: alkaline phosphatase
Arl6ip5: ADP-ribosylation-like factor 6 interacting protein 5
ATF4: activating transcription factor 4
BMP-2: bone morphogenetic protein 2
CaM: calmodulin
CaMKII: Ca2+/calmodulin-dependent protein kinase II
Chop: CCAAT/Enhancer-binding protein homologous protein
Dex: dexamethasone
ER: endoplasmic reticulum
IP3Rs: inositol trisphosphate 3 receptors
ISO: isoproterenol hydrochloride
MAR: mineral apposition rate
POBs: primary calvarial osteoblasts
PTH: parathyroid hormone
RANKL: receptor activator of nuclear factor-*κ*B ligand
TG: thapsigargin
TGF*β*: transforming growth factor *β*
TRAP: tartrate resistant acid phosphatase
UPR: unfolded protein response
*μ*-CT: micro-computed tomography
