# Correction to: Deficiency of osteoblastic Arl6ip5 impaired osteoblast differentiation and enhanced osteoclastogenesis via disturbance of ER calcium homeostasis and induction of ER stress-mediated apoptosis

**DOI:** 10.1038/s41419-024-06858-5

**Published:** 2024-07-31

**Authors:** Y. Wu, M. Yang, J. Fan, Y. Peng, L. Deng, Y. Ding, R. Yang, J. Zhou, D. Miao, Q. Fu

**Affiliations:** 1 Key Laboratory of Nuclear Medicine, Ministry of Health, Jiangsu Key Laboratory of Molecular Nuclear Medicine, Jiangsu Institute of Nuclear Medicine, Wuxi, China; 2 Department of Radiotherapy, The First Affiliated Hospital of Nanjing Medical University, Nanjing, China; 3 Department of Molecular Cell Biology and Toxicology, School of Public Health, Nanjing Medical University, Nanjing, China; 4 State Key Laboratory of Reproductive Medicine, The Research Center for Bone and Stem Cells, Department of Anatomy, Histology and Embryology, Nanjing, China

Correction to: *Cell Death & Disease* 10.1038/cddis.2014.427, published online 16 October 2014

In the original published version of this article, the authors unintentionally misplaced images in Figure 3d and wish to amend this by providing the correct image of the 12d differentiation of Arl6ipΔ2/Δ2 cell. The authors apologize for the errors.
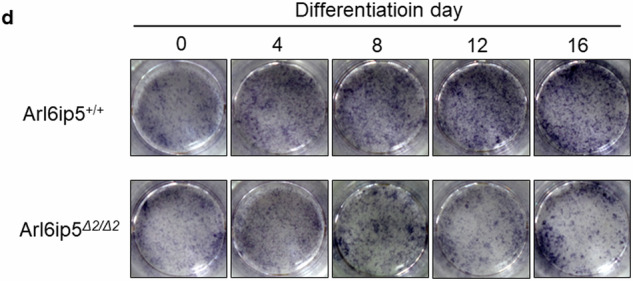


### Supplementary information

Original data-1

Original data-2

