# Supplementary material for: Deficiency of osteoblastic Arl6ip5 impaired osteoblast differentiation and enhanced osteoclastogenesis via disturbance of ER calcium homeostasis and induction of ER stress-mediated apoptosis
Source: Cell Death Dis. 2014 Oct 16;5(10):e1464–. doi: 10.1038/cddis.2014.427 (PMC4237252; doi:10.1038/cddis.2014.427)
Supplement: Supplementary Figures [file cddis2014427x1.doc]

**Supplementary materials**

**Supplementary Figure Legends**

**Supplementary Fig.S1** The basic expression of Arl6ip5 in bone tissues. Arl6ip5 mRNA levels was analyzed in liver, kidney and bone tissues from wild type C57BL6/J mice (n=4) with Q-PCR analysis.

**Supplementary Fig.S2** Arl6ip5 deficiency mice (Arl6ip5*^Δ2/Δ2^*) showed decrease of bone mineral density (BMD). The tibia (A) and Lumbar vertebra (L3-5) (B) from 16 weeks old Arl6ip5*^Δ2/Δ2^* (n=12) and their wild type littermates (Arl6ip5^+/+^) (n=10) were analyzed with μ-CT and BMD was calculated. Each bar represents mean±SEM. *, *Student’s t-test*, *P*<0.05.

**Supplementary Fig.S3** Mineral apposition rate (MAR) of tibia trabecular bone in Arl6ip5^+/+^ (n = 6) and Arl6ip5*^Δ2/Δ2^* mice (n = 6), which based on the double calcein staining. Scale bar, 100 μm. Bar represents mean±SEM. *, *Student’s t-test*, *P*<0.05.

**Supplementary Fig.S4** Arl6ip5 mRNA in adherent bone marrow cells (precursors of osteoblasts, Ob.P) and non-adherent bone marrow cells (precursors of osteoclasts, Oc.P) were compared with Q-PCR analysis. n=3.

**Supplementary Fig.S5** The effect of siRNA treatment on mRNA expression of Arl6ip5 in UAMS-32 cell. Cells were transfected with the negative control siRNA (NC-siRNA) and Arl6ip5-siRNA for 72 hours, then the expression of Arl6ip5 were analyzed with Q-PCR. n=3.

**Supplementary Fig.S6** ATP-stimulated [Ca^2+^]_i_ in a time course was analyzed in Arl6ip5^+/+^ and Arl6ip5*^Δ2/Δ2^* POB. Representative results from four independent experiments were shown.

**Supplementary Fig.S7** Arl6ip5 regulated basal ER Ca^2+^. As the Ca^2+^ pool released by addition of the Ca^2+^-ionophore ionomycin. 10 μM ionomycin + 2.5mM EGTA were added to cells and the increase in cytosolic Ca^2+^ was measured (Δpeak). n=3. *, *Student’s t test*, P<0.05.

**Supplementary Fig.S8** Expression of ATF4, Bip, Grp94, Pdia3, Chop and Gadd34 mRNAs in the tibias from Arl6ip5*^Δ2/Δ2^* (n = 6) and Arl6ip5^+/+^ (n = 6) mice. In all panels, Scale bar represents mean±SEM, *P < 0.05, **P < 0.01. All P values were based on *Student’s t test*.

**Supplementary Fig.S9** Expression of ATF4, Bip, Grp94, P4hb, Pdia3, Chop and Gadd34 mRNAs in the UAMS-32 cells with Arl6ip5-siRNA (n=3) and NC-siRNA (n=3) treated. In all panels, Scale bar represents mean±SEM, *P < 0.05, **P < 0.01. All P values were based on *Student’s t test*.

**Supplementary Fig.S10** Arl6ip5 deficiency induced osteoblast apoptosis in vivo. (A-B) TUNEL staining for tibia sections from 4 moth old Arl6ip5^+/+^ and Arl6ip5*^Δ2/Δ2^* mice. Representative images were shown. The areas selected by rectangle were enlarge to show the positive staining osteoblast (O.b) and osteocyte (O.c), bar=100μm. (B) The proportion of positive staining osteoblasts was calculated. (C) Expression of Bcl-2 mRNA in the tibias from Arl6ip5*^Δ2/Δ2^* (n = 6) and Arl6ip5^+/+^ (n = 6) mice. Scale bar represents mean±SEM, **P < 0.01. All P values were based on *Student’s t test*.

**Supplementary Fig.S11** Expression of Bim-1, Puma and Bcl-2 mRNAs in the UAMS-32 cells with Arl6ip5-siRNA (n=3) and NC-siRNA (n=3) treated. In all panels, Scale bar represents mean±SEM, *P < 0.05, **P < 0.01. All P values were based on *Student’s t test*.

**Supplementary Fig.S12** Expression of Trib3, one downstream target gene of Chop in the UAMS-32 cells with Arl6ip5-siRNA (n=3) and NC-siRNA (n=3) treated. *P < 0.05, *Student’s t test*.

**Supplementary Fig.S13** Calcium signaling involves in the regulation of Arl6ip5 on osteoblast proliferation. (A) siRNA treated UAMS-32 cells received extracellular CaCl_2_ (20mM) treatment and for MTT analysis. *. NC-siRNA+Ctrl *vs* Arl6ip5-siRNA+Ctrl, P<0.05; #, Arl6ip5-siRNA+CaCl_2_ *vs* Arl6ip5-siRNA+Ctrl, P<0.05. (B) c-Fos mRNA level in HA-Arl6ip5 unexpressed UAMS-32 cells with or without KN-93 (1μM) and STO-609 (1μM) treatment. *, P<0.05. In all panels, n=4. Error bars represent the mean±SEM.

**Supplementary Fig.S14** Osteoblastic Arl6ip5 regulated RANKL expression. Q-PCR analyzed the RANKL transcription induced by 1,25-(OH)2-VitD3 treatment in UAMS-32 cells with Arl6ip5-siRNA treatment. n=3 and *P < 0.05 was based on *Student’s t test*.

**Supplementary Fig.S15** Osteoblastic Arl6ip5 regulated RANKL expression. soluble RANKL (sRANKL) in culture medium of Arl6ip5-siRNA treated UAMS-32 cells (A) and Arl6ip5^Δ2/Δ2^ POBs (B) were detected with ELISA. Cells were treated with PTH (100nM) to induced RANKL expression (A and B). In both panels, each bar represents mean±SEM (n = 3-4). *P < 0.05. All P values were based on *Anova*.

**Supplementary Fig.S16** Bone resorption in cocultures of Arl6ip5^+/+^ or Arl6ip5^Δ2/Δ2^ primary osteoblast (POBs) with Raw264.7 cells as osteoclast (OC) precursors, treated with PTH (100nM). Representative images were shown. Arrows indicated the resorption areas. Histograms quantify the average proportion of resorption pit formation areas. Bar represents mean±SEM (n = 3). **P < 0.01 by *Student’s t test*.

**Supplementary Fig.S17** cAMP-PKA pathway was not involved in the RANKL expression mediated by Arl6ip5 deficiency. UAMS-32 cells were firstly received Arl6ip5-siRNA treatment for 48 hours, then the medium was changed and H-89, the inhibitor of PKA signaling, was supplemented with final concentration 20μM for 24 hours. Q-PCR was used to analyze the RANKL expression. The mRNA level of Arl6ip5 was showed under the panel. n=3 and *P < 0.05; **P < 0.01. All P values were based on *Anova*.

**Supplementary Fig.S18** ATF4 was not involved in the RANKL expression mediated by Arl6ip5 deficiency. UAMS-32 cells were firstly received ATF4-siRNA treatment for 24 hours, then medium was changed and the cells received Arl6ip5-siRNA or its control for 72 hours. Q-PCR was used to analyze the Arl6ip5, ATF4 and RANKL expression. The mRNA level of Arl6ip5 was showed under the panel. n=3 and *P < 0.05; **P < 0.01. All P values were based on *Anova*.


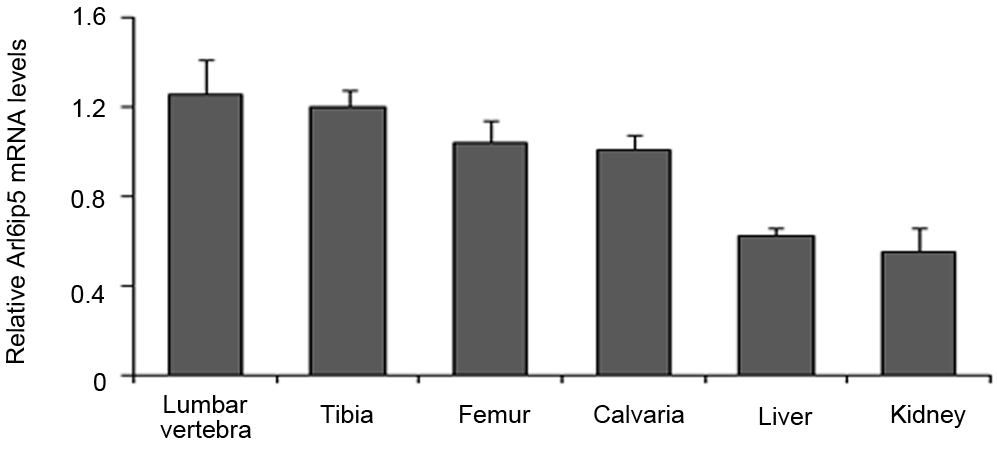


Supplementary Fig.S1


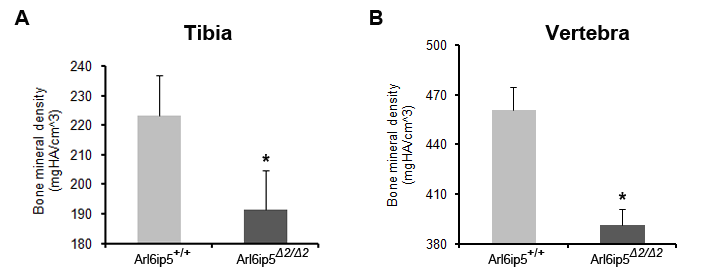


Supplementary Fig.S2


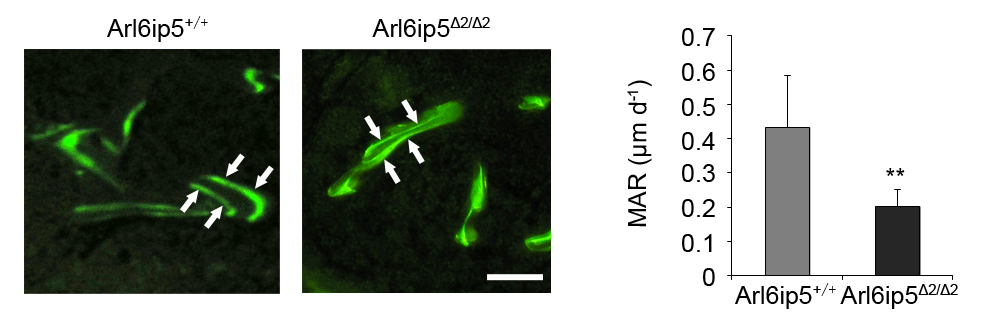


Supplementary Fig.S3


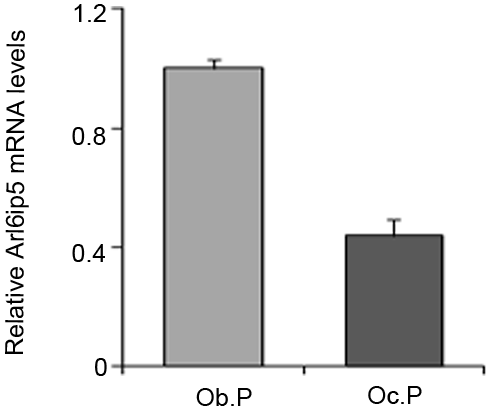


Supplementary Fig.S4


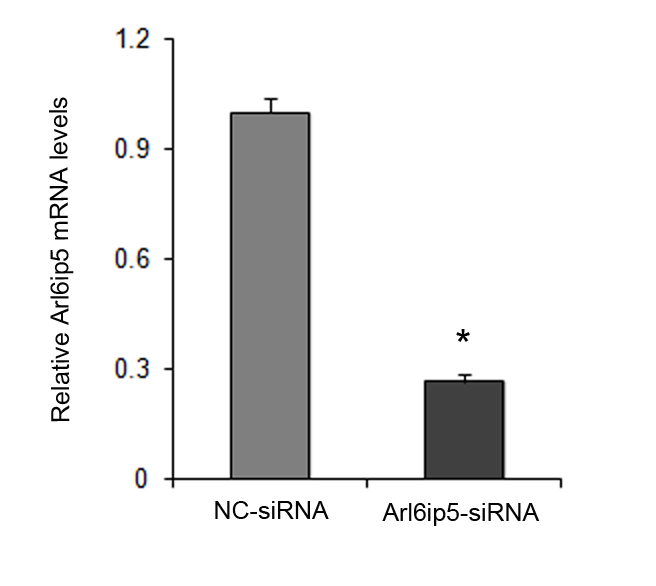


Supplementary Fig.S5


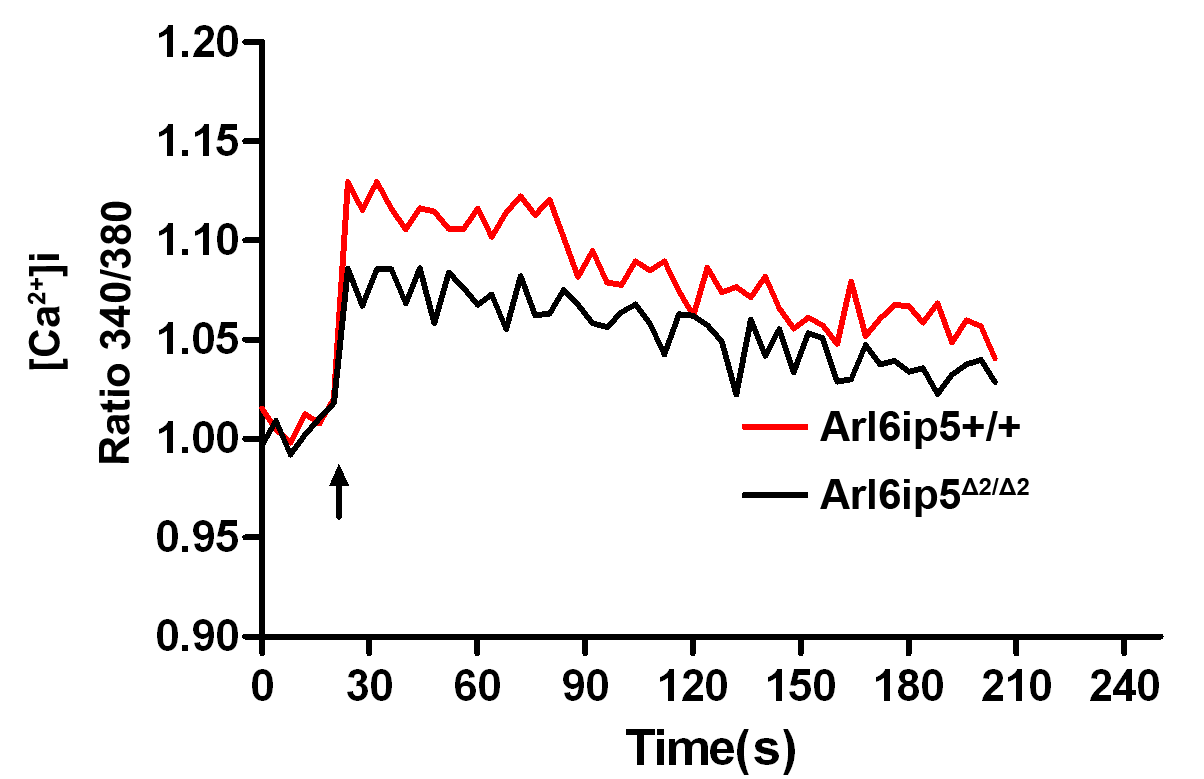


Supplementary Fig.S6


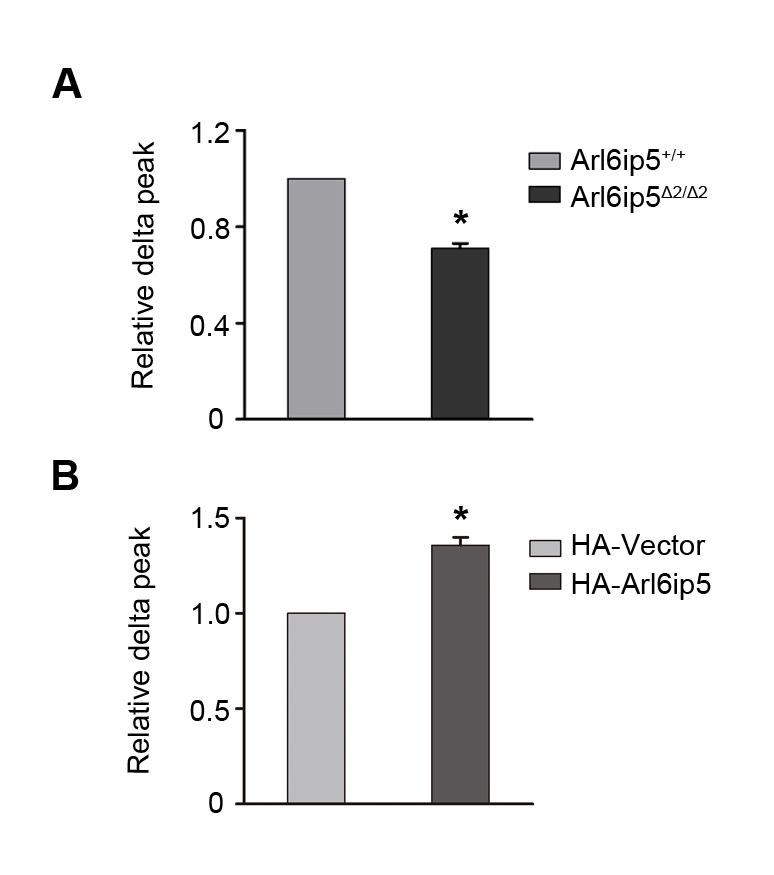


Supplementary Fig.S7


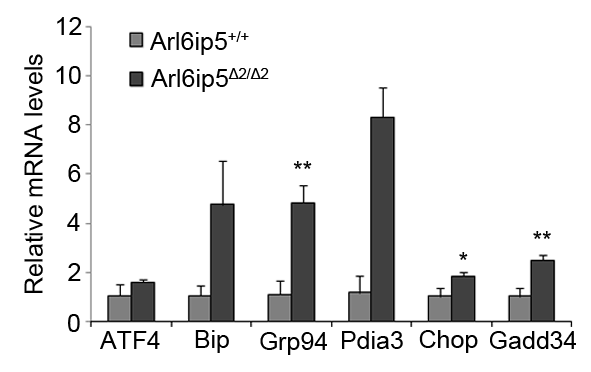


Supplementary Fig.S8


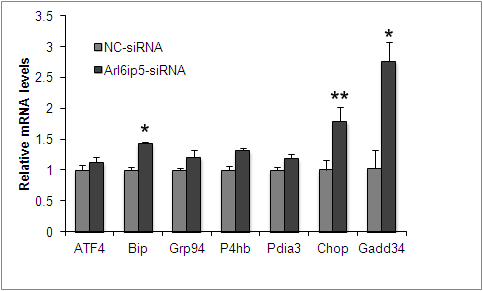


Supplementary Fig.S9


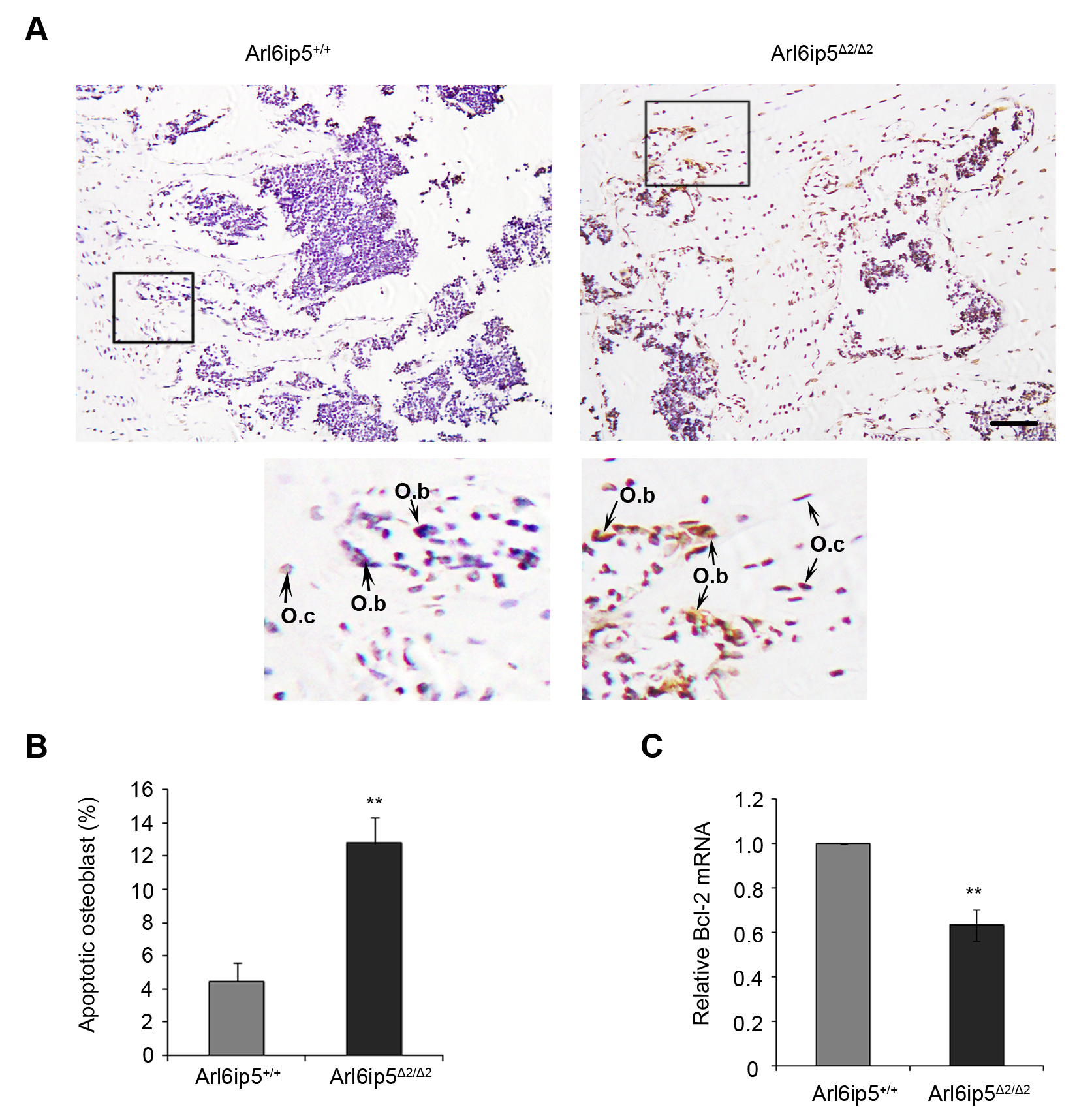


Supplementary Fig.S10


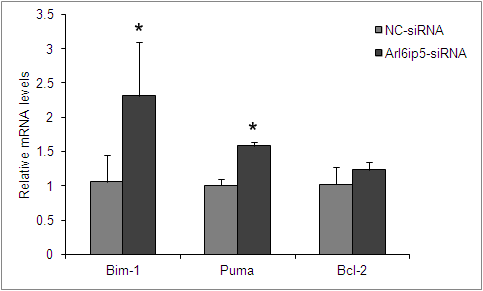


Supplementary Fig.S11


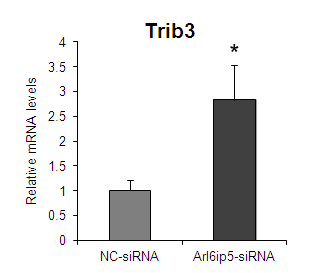


Supplementary Fig.S12


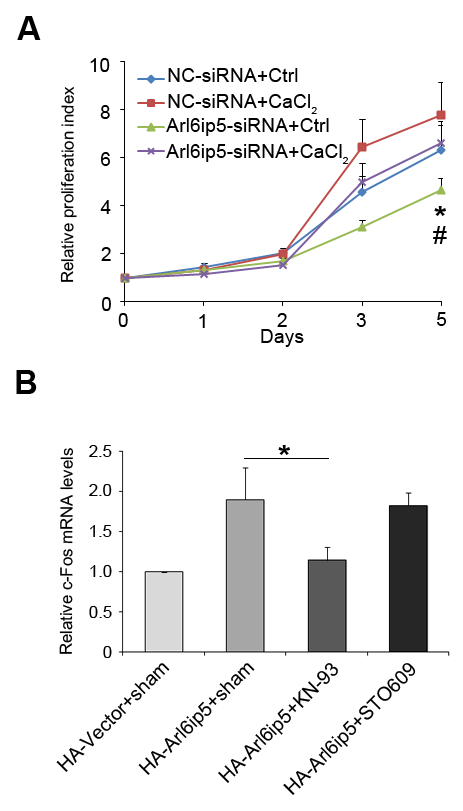


Supplementary Fig.S13


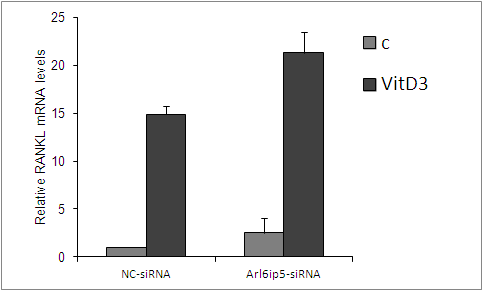


Supplementary Fig.S14


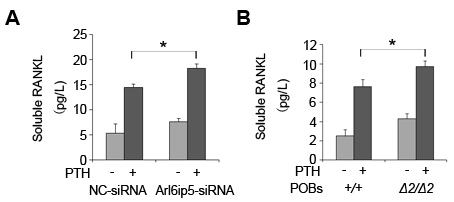


Supplementary Fig.S15


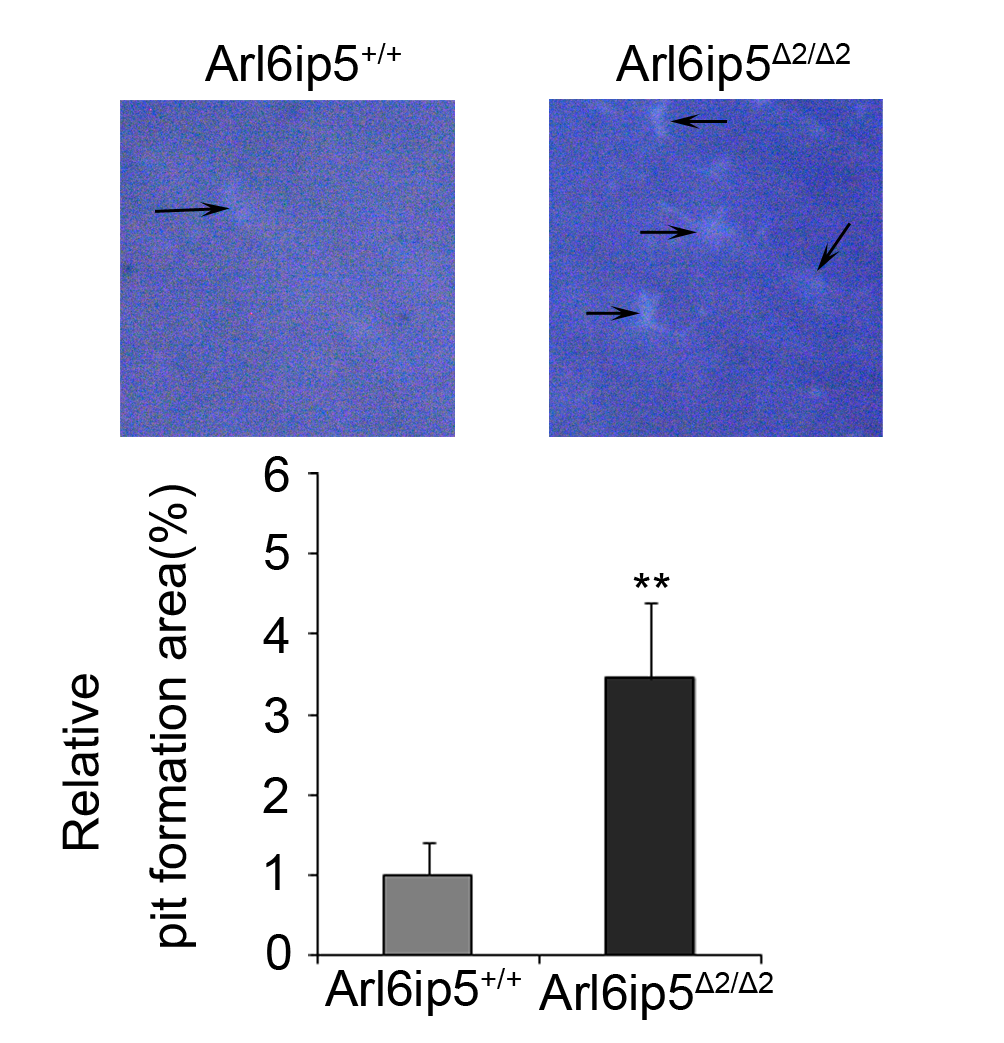


Supplementary Fig.S16


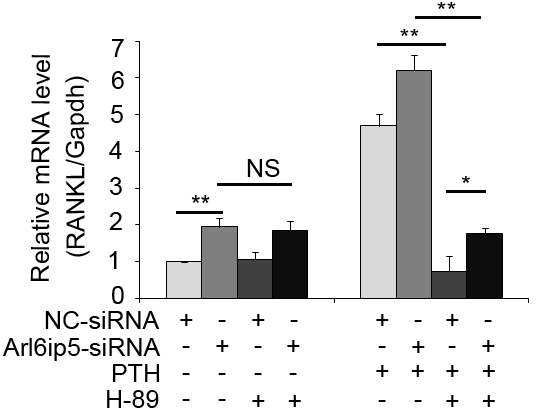


Supplementary Fig.S17


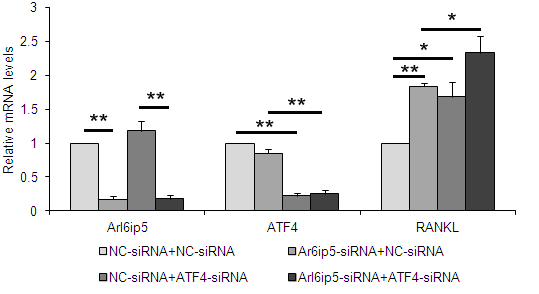


Supplementary Fig.S18
