# Supplementary figures and images for: Correction to: Deficiency of osteoblastic Arl6ip5 impaired osteoblast differentiation and enhanced osteoclastogenesis via disturbance of ER calcium homeostasis and induction of ER stress-mediated apoptosis
Source: Cell Death Dis. 2024 Jul 31;15(7):550. doi: 10.1038/s41419-024-06858-5 (PMC11291709; doi:10.1038/s41419-024-06858-5)

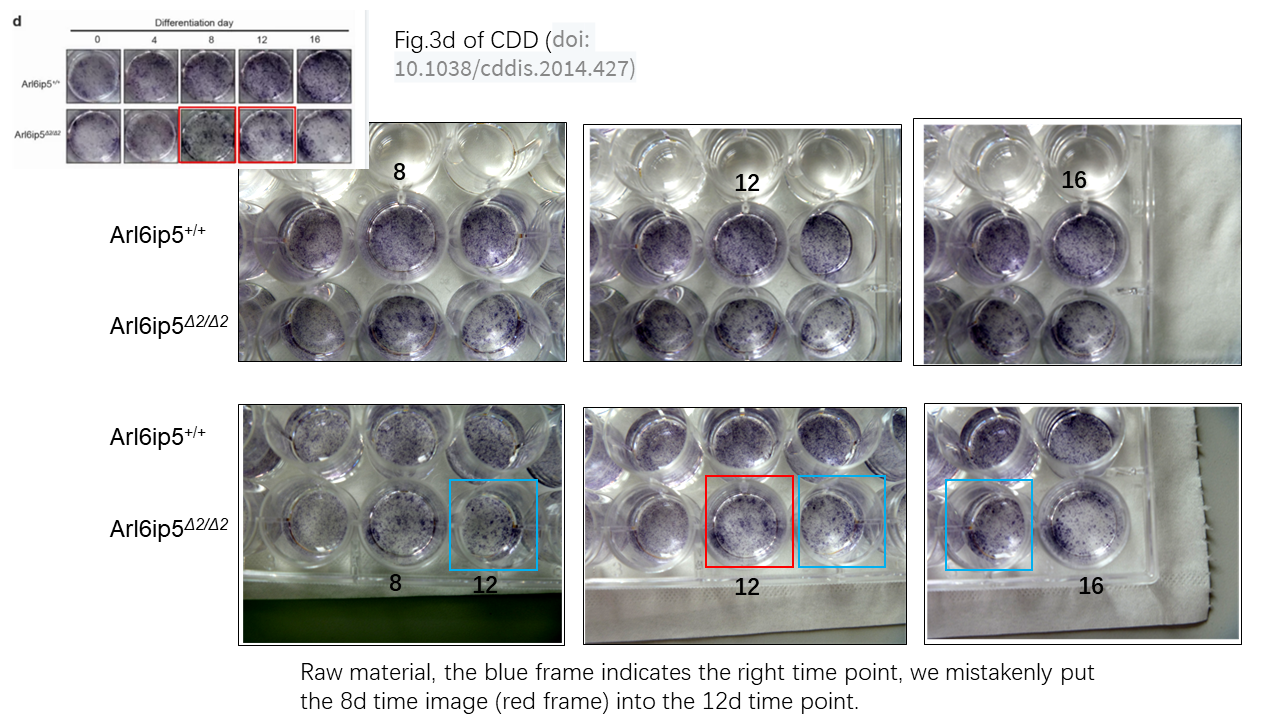

Supplement: Supplementary file 1 — Original data-1 [file 41419_2024_6858_MOESM1_ESM.tif]

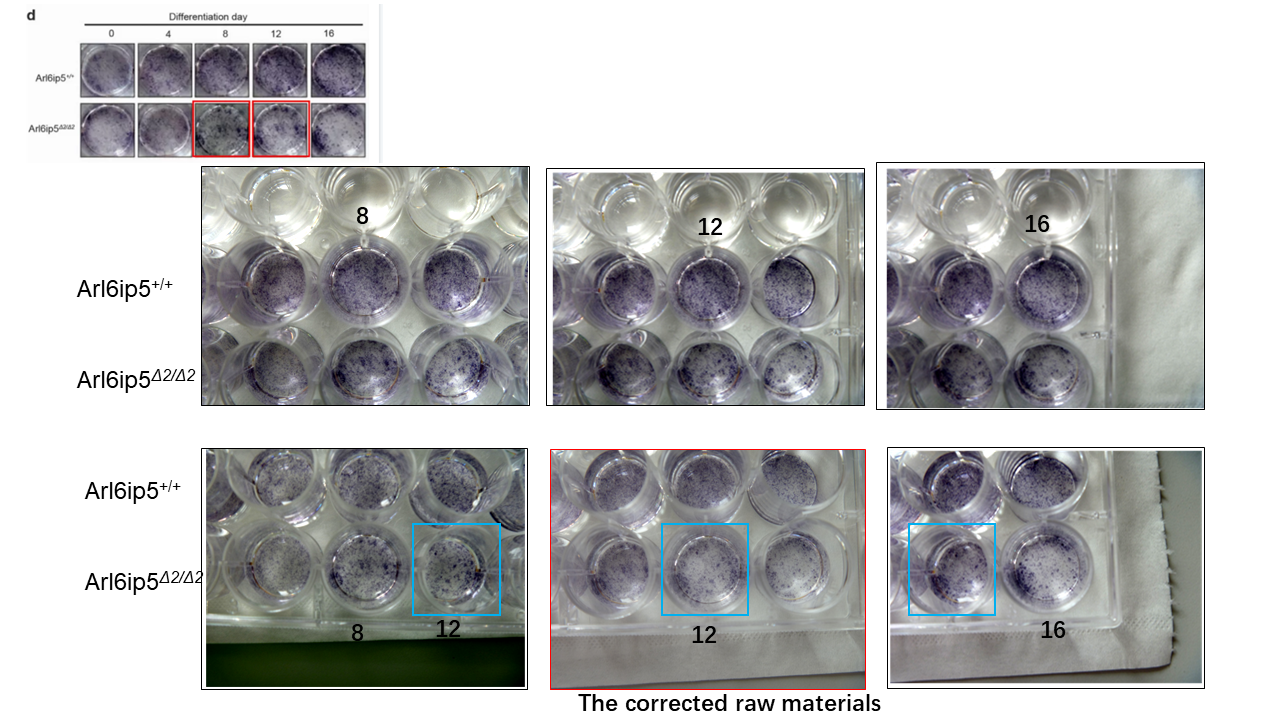

Supplement: Supplementary file 2 — Original data-2 [file 41419_2024_6858_MOESM2_ESM.tif]
